# Integrated bioinformatics and statistical approaches to explore molecular biomarkers for breast cancer diagnosis, prognosis and therapies

**DOI:** 10.1371/journal.pone.0268967

**Published:** 2022-05-26

**Authors:** Md. Shahin Alam, Adiba Sultana, Md. Selim Reza, Md Amanullah, Syed Rashel Kabir, Md. Nurul Haque Mollah

**Affiliations:** 1 Bioinformatics Lab (Dry), Department of Statistics, University of Rajshahi, Rajshahi, Bangladesh; 2 Center for Systems Biology, Soochow University, Suzhou, China; 3 Department of Respiratory Medicine, Sir Run Run Shaw Hospital and Institute of Translational Medicine, Zhejiang University School of Medicine, Hangzhou, Zhejiang, China; 4 Department of Biochemistry and Molecular Biology, Rajshahi University, Rajshahi, Bangladesh; Tanta University Faculty of Medicine, EGYPT

## Abstract

Integrated bioinformatics and statistical approaches are now playing the vital role in identifying potential molecular biomarkers more accurately in presence of huge number of alternatives for disease diagnosis, prognosis and therapies by reducing time and cost compared to the wet-lab based experimental procedures. Breast cancer (BC) is one of the leading causes of cancer related deaths for women worldwide. Several dry-lab and wet-lab based studies have identified different sets of molecular biomarkers for BC. But they did not compare their results to each other so much either computationally or experimentally. In this study, an attempt was made to propose a set of molecular biomarkers that might be more effective for BC diagnosis, prognosis and therapies, by using the integrated bioinformatics and statistical approaches. At first, we identified 190 differentially expressed genes (DEGs) between BC and control samples by using the statistical LIMMA approach. Then we identified 13 DEGs (*AKR1C1*, *IRF9*, *OAS1*, *OAS3*, *SLCO2A1*, *NT5E*, *NQO1*, *ANGPT1*, *FN1*, *ATF6B*, *HPGD*, *BCL11A*, and *TP53INP1*) as the key genes (KGs) by protein-protein interaction (PPI) network analysis. Then we investigated the pathogenetic processes of DEGs highlighting KGs by GO terms and KEGG pathway enrichment analysis. Moreover, we disclosed the transcriptional and post-transcriptional regulatory factors of KGs by their interaction network analysis with the transcription factors (TFs) and micro-RNAs. Both supervised and unsupervised learning’s including multivariate survival analysis results confirmed the strong prognostic power of the proposed KGs. Finally, we suggested KGs-guided computationally more effective seven candidate drugs (NVP-BHG712, Nilotinib, GSK2126458, YM201636, TG-02, CX-5461, AP-24534) compared to other published drugs by cross-validation with the state-of-the-art alternatives top-ranked independent receptor proteins. Thus, our findings might be played a vital role in breast cancer diagnosis, prognosis and therapies.

## Introduction

Breast cancer (BC) is one of the most common types of invasive cancers among women according to the World Health Organization (WHO), which affected around 2.3 million women in 2020. It is also the cause of large number of cancer-related deaths among women worldwide [1]. Symptoms of BC include a change in breast shape, dimpling of the skin, nipple discharge, or a red scaly patch of skin, and a lump in the breast [2]. Based on the existing treatment facilities, the average 5-year survival rate with BC is 86%, but BC with distant metastasis, the average 5-year survival rate drops down to 28% [3]. Thus, the performance of existing therapeutic treatments on BC is not yet reach to the satisfactory level. Therefore, in-depth molecular research is essential to explore BC causing more effective biomarkers and candidate drugs.

However, new drug discovery is a tremendous challenging, time consuming and expensive task. The main challenges are to explore drug target proteins (receptors) responsible for diseases and drug agents (small molecules) that can reduce the diseases by the interaction with the target proteins. Genomic biomarkers induced proteins are considered as the key receptors. Transcriptomics analysis is a widely used popular approach to explore genomic biomarkers [4–8]. The repurposing of existing drugs for other diseases could reduce the time and cost compared to de novo drug development. By this time, several authors suggested several sets of genomic biomarkers to explore molecular mechanisms and pathogenetic processes of BC [9–17]. Some of them also suggested candidate drugs for the treatment against BC [18, 19]. However, their published data did not display any common set of receptors and/or drugs, and so far, none of them yet investigated the resistance of their suggested drugs against the independent receptors proposed by others. Obviously, a question may be raised, how a drug can be effective globally for all peoples around the world. Therefore, in this study, our main objectives are (i) computational identification of genomic biomarkers (drug targets) for BC highlighting their functions, pathways and regulatory factors, (ii) exploring genomic biomarker guided candidate drugs for the treatment against BC, and (iii) In-silico validation on the resistance performance of the proposed candidate drugs against the state-of-the-art alternatives top-ranked independent receptors associated with BC published by others.

## Materials and methods

**Fig 1:**
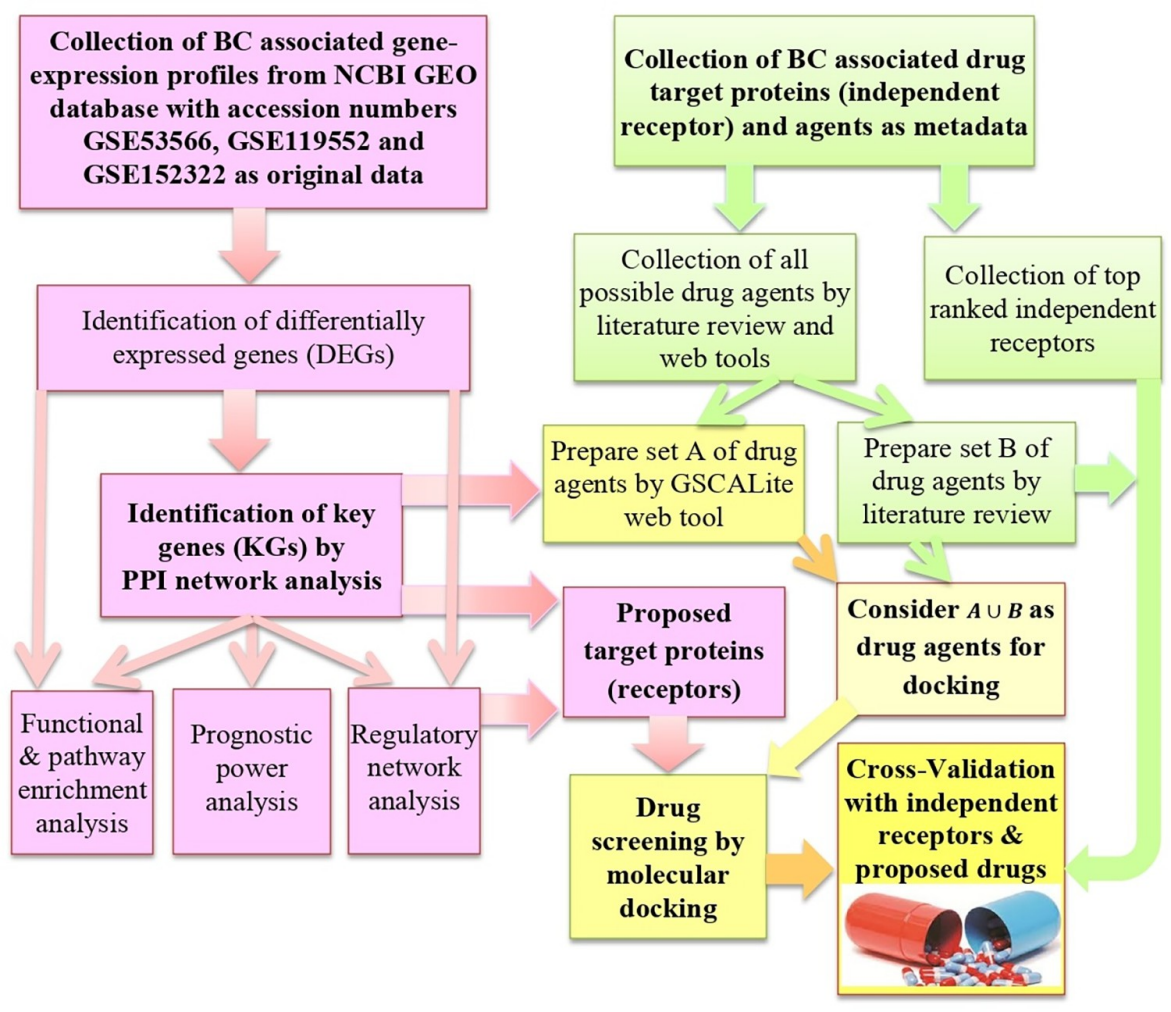
The pipeline of this study.

To reach the goal of this study, we considered both raw-data (gene expression profiles) and meta-data associated with BC. Integrated bioinformatics and statistical approaches were used to analyze the datasets to explore KGs highlighting their functions, pathways, regulatory factors, prognosis power and repurposable drugs. The pipeline of this study is given in **Fig 1**.

### Data sources and descriptions

#### Collection of gene expression profiles for exploring KGs

The microarray gene expressions profile dataset with accession number GSE53566 [20] was downloaded from the National Center of Biotechnology Information (NCBI) Gene Expression Omnibus (GEO) database. The dataset was generated based on two different BC cell lines (BT-20 and MDA-MB-231), either overexpression (BT-20) or knock-down (MDA-MB-231). Untreated cell lines served as controls. The whole genome expression profiles were consisted of 8 treated (case) and 8 control samples with 41078 probes. To investigate the prognostic performance of KGs unbiasedly, we collected two independent microarray gene expression datasets with accession numbers GSE119552 [21] and GSE152322 [22], respectively. More information about these datasets were given in **S1 Table in S1 File**.

#### Collection of meta-drug agents for exploring candidate drugs

We collected meta-drug agents (small molecules) from the online database GSCALite [23] by the significant correlation with our proposed target proteins (set-A) and published articles (set-B) to explore candidate drugs (set-C) by molecular docking with our proposed target proteins (genomic biomarkers). Both A and B sets of meta-drug agents were given in the supplementary file (**S2 Table in S1 File**).

#### Collection of independent meta-receptors for cross-validation with the proposed drugs

To select the top-ranked hub-genes (meta-receptors) associated with BC; we reviewed 78 published articles and selected the top-ranked 13 target proteins as the meta-receptors (**S3 Table in S1 File**).

### Identification of DEGs

To identify DEGs between BC and normal conditions, we considered the linear models for microarray (LIMMA) data analysis suggested by Smyth (2004) [24], which can be written as

$$y_{g}=X\alpha_{g}+\in_{g}$$ (1)

Where y_g_ = (y_g1_, y_g2_,…,y_gn_)^/^ is the vector of expressions (responses) for *g*th gene with n = n_1_+n_2_ samples (g = 1, 2, …, m), X is an n×2 design matrix, α_g_ = (α_g1_, α_g2_)^/^ is 2×1 vector (2<n) of effects for two different groups of n samples and the error vector $\in_{g}∼N(0,W_{g}\sigma_{g}^{2})$. Here W_g_ is a positive definite weight matrix. We want to test the null hypothesis (H_0_): α_g1_ = α_g2_ = > γ_g_ = (α_g1_−α_g2_) = 0 (that is, gth gene is equally expressed gene (EEG) in both case and control groups) against the alternative hypothesis (H_1_): α_g1_≠α_g2_ = > γ_g_≠0 (that is, *g*th gene is differentially expressed gene (DEG) between case and control groups). To test H_0_ against H_1_, the moderated t-statistic was formulated by hybridizing the classical and Bayesian approaches in which the posterior variance is substituted into to the classical t-statistic in place of the classical sample variance. The moderated t-statistic was defined as,

$${\tilde{t}}_{g}=\frac{{\hat{\gamma }}_{g}-\gamma_{g}}{{\tilde{s}}_{g}\sqrt{\delta_{g}}}$$ (2)

which follows t-distribution with d_g_+d_0_ degrees of freedom under H_0_.

Adjusted *P-*value based on the moderated *t*-statistic and the average of log2 fold-change (aLog_2_FC) values of treatment group with respect to the control group were used to select DEGs or EEGs as follows

$${DEG}_{g}=\left\{ \begin{array}{l} DEG\;(Upregulated),\;if\;adj.p.value<0.01\;and\;{aLog}_{2}{FC}_{g}>+1.0\\ EEG,\;if\;adj.p.value<0.01\;and\;-1.0<{aLog}_{2}{FC}_{g}<1.0\\ DEG\;(Downregulated),\;if\;adj.p.value<0.01\;and\;{aLog}_{2}{FC}_{g}<-1.0 \end{array}\right.$$ (3)

where

$$a{Log}_{2}{FC}_{g}=\left\{ \begin{array}{l} \frac{1}{n_{1}}\sum_{i}^{n_{1}}{log}_{2}(y_{gi}^{T})-\frac{1}{n_{2}}\sum_{j}^{n_{2}}{log}_{2}(y_{gj}^{C}),\;if\;n_{1}\neq n_{2}\\ \frac{1}{n}\sum_{i}^{n}{log}_{2}(\frac{y_{gi}^{T}}{y_{gi}^{C}}),\;if\;n_{1}=n_{2}=n. \end{array}\right.$$

Here $y_{gi}^{T}$ and $y_{gj}^{C}$ are the expressions for the *g*th gene with the *i*th treatment and *j*th control samples, respectively. We implemented the limma R-package [25] for calculating the *P*-values and aLog_2_FC values to select the DEGs, significantly.

### Construction of PPI network of DEGs

Protein-protein interaction (PPI) network was constructed to identify key-genes (KGs). The online STRING-v11 database [26] was used to construct the PPI network of DEGs. The STRING database provides critical assessment and integration of protein interactions, including direct (physical) and indirect (functional) associations. To construct PPI network, the distance ‘D’ between pair of proteins (u,v) is calculated as

$$D(u,v)=\frac{2|{N_{u}⋂N}_{v}|}{{|N}_{u}|+{|N}_{v}|}$$ (4)

Where *N*_*u*_ is the neighbor set of *u* and *N*_*v*_ is the neighbor set of *v*. Cytoscape plug-in cytoHubba is used to rank the nodes of PPI network for identifying KGs in the network [27, 28]. In the present study five topological methods including Degree [29], BottleNeck [30], Betweenness [31], Stress [32], and Clustering Coefficient was utilized to identify KGs.

### GO terms and KEGG pathway enrichment analysis of DEGs highlighting KGs

The GO (Gene Ontology) functions and KEGG (Kyoto Encyclopedia of Genes and Genomes) pathway enrichment analysis were performed to understand the pathogenetic processes and pathways of DEGs highlighting KGs. The GO terms have three categories: Biological Process (BP), Cellular Component (CC), and Molecular Function (MF). To explore the significantly enriched GO terms and KEGG pathways by DEGs including KGs, let *S*_*i*_ is the annotated gene-set corresponding to *i*^th^ type of biological functions or pathways given in the database and *M*_*i*_ is the number of genes in *S*_*i*_ (*i* = 1, 2,…,*r*); *N* is the total number of annotated genes those construct the entire combine set $S=\overset{r}{\underset{i=1}{⋃}}S_{i}=S_{i}⋃S_{i}^{c}$ such that $N\leq \sum_{i=1}^{r}M_{i}$; where $S_{i}^{c}$ is the complement set of *S*_*i*_. Again let *n* is the total number of DEGs of interest and *k*_*i*_ is the number of DEGs belonging to the annotated gene-set *S*_*i*_. This problem is summarized by the following contingency table (**Table 1**).

**Table 1:** Contingency table.

Annotated Gene-sets	DEGs (proposed)	EEGs (proposed)	Marginal total (Annotated)
*i*^th^ GO term/KEGG pathway (*S*_*i*_)	*k*_*i*_	*M*_*i*_ *—k*_*i*_	*M*_*i*_
Complement of *S*_*i*_ ($S_{i}^{c}$)	*n—k*_*i*_	*N—M*_*i*_ *−n + k*_*i*_	*N—M*_*i*_
Marginal total	*n*	*N—n*	*N* (Grand total)

To find the significantly enriched GO terms and KEGG pathways by our proposed DEGs, the *P*-value was calculated by the Fisher exact test statistic based on hypergeometric distribution. We used DAVID online tool (version 6.8) to perform Fisher exact test [33].

### Regulatory network analysis of KGs

To identify key transcription factors (TFs) as the transcriptional regulators of KGs, the TFs-KGs interaction network was constructed using the publicly available database JASPAR [34]. The interaction network was generated using NetworkAnalyst [35]. To identify key microRNAs (miRNAs) as the post-transcriptional regulators of KGs, the KGs-miRNAs interaction network was constructed by using the publicly available online tool miRNet 2.0 [36]. The top degree miRNAs were selected from the network and considered them as key miRNAs.

### Prognostic power analysis of KGs

To investigate the prognostic power of KGs, we performed cluster analysis, survival analysis and developed two prediction models using random forest (RF) and support vector machine (SVM) classifiers. The survival curve and ROC curve were used to assess the prognosis performance. The online SurvExpress computational tool [37] was used to produce survival curve. The R-packages ‘gplots’ and ‘ROCR’ were used to produce heatmap and ROC curve, respectively.

### Molecular docking simulation for exploring candidate drugs

To propose *in-silico* validated effective drugs for the treatment against BC, we employed molecular docking simulation between the target receptor proteins and drug agents. We considered our proposed KGs based hub-proteins and associated TFs proteins as the drug target receptor proteins and meta-drug agents collected from online databases and published articles for docking analysis. The molecular docking simulation requires 3-Dimensional (3D) structures of both receptor proteins and candidate drugs. We downloaded 3D structure of all targeted receptor proteins from Protein Data Bank (PDB) [38] and SWISS-MODEL [39]. The 3D structures of drug agents were downloaded from PubChem and DrugBank database [40, 41]. The 3D structure of the target proteins was visualized using Discovery Studio Visualizer 2020 and the water molecules, co-crystal ligands which were bound to the protein were removed. Further, the protein was prepared using USCF Chimera and Autodock vina ^136^^51^ in PyRx open source software by adding charges and minimizing the energy of the protein and subsequently converting it to pdbqt format [42–44]. The exhaustiveness parameter was set to 8. The Protein-Ligand Interaction Profiler (PLIP) web service [45] and PyMol was used to analyze the docked complexes for surface complexes, types and distances of non-covalent bonds. Let *A*_*ij*_ denotes the binding affinity between *i*^th^ target protein (*i* = 1, 2, …, *m*) and *j*^th^ drug agent (*j* = 1, 2,…, *n*). Then target proteins are ordered according to the descending order of row means $\sum_{j=1}^{n}A_{ij/}/m$, *j* = 1,2,…,*m*, and drug agents are ordered according to the descending order of column means $\sum_{i=1}^{m}A_{ij}/n$, *j* = 1,2,…,*n*, to select the top ranking few drug agents as the candidate drugs. Then we validated the proposed candidate drugs by molecular docking simulation with the top ordered independent receptors associated with BC published by others.

## Results

### Identification of DEGs

**Fig 2:**
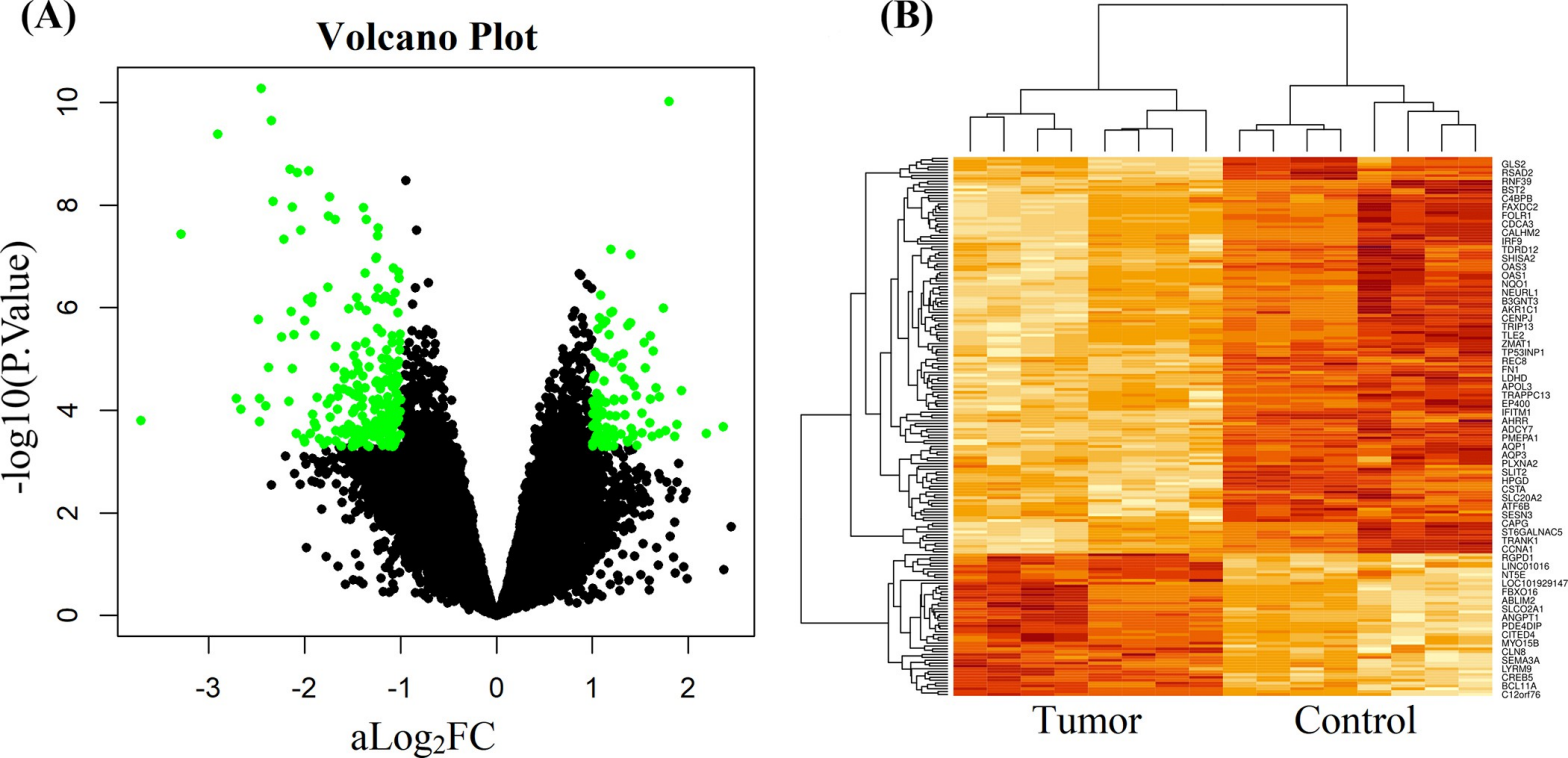
**(A)** Volcano plot of–log_10_(*P*-value) against log_2_FC values to display significantly upregulated and downregulated DEGs. **(B)** Heatmap of significantly upregulated and downregulated DEGs to observe the clustering performance of tumor and control groups by hierarchical clustering approach.

We identified 190 DEGs, including 138 downregulated and 52 upregulated genes (**S1 Table in S2 File**) in BC tissue, using adj.*P*.Val < 0.01 and logFC > 1 as the threshold for upregulated DEGs, and adj.*P*.Val < 0.01 and logFC < -1 for downregulated DEGs. The upregulated and downregulated DEGs were displayed on the right and left sides respectively in the volcano plot by the green color in **Fig 2A**. A heatmap was constructed to show the clustering performance of case and control samples by the up and down regulated DEGs in **Fig 2B**. We observed that both DEGs and samples separated each other between their contrast groups accurately.

### Identification of key genes (KGs) from DEGs

**Fig 3:**
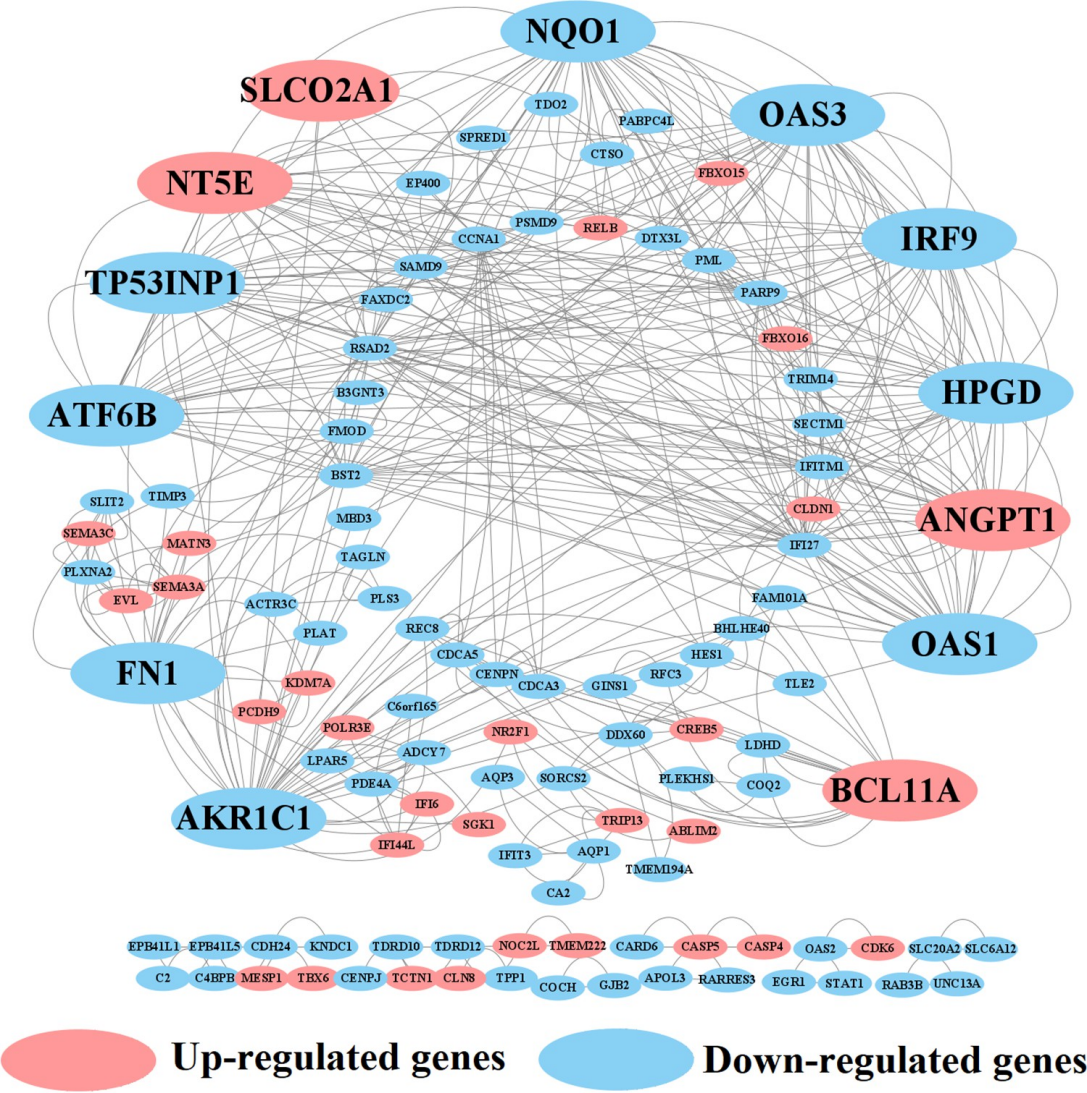
Protein-protein interaction (PPI) network of DEGs to select the key genes (KGs). Blue color indicates downregulated and pink color indicates upregulated DEGs, big size and octagon shape indicate the KGs.

To identify KGs, the PPI network of DEGs was constructed which includes 180 nodes and 218 edges, with an average node degree 2.42 and *P*-value < 1.0e-16. In the PPI network, pink color indicates up regulated and blue color indicates down regulated DEGs, big size and octagon shape indicate KGs (see **Fig 3**). We used five topological measures (Degree, BottleNeck, Betweenness, Stress and Clustering Coefficient) to select top-ranked 13 KGs that are *AKR1C1*, *IRF9*, *OAS1*, *OAS3*, *SLCO2A1*, *NT5E*, *NQO1*, *ANGPT1*, *FN1*, *ATF6B*, *HPGD*, *BCL11A*, and *TP53INP1*, where 4 KGs (*SLCO2A1*, *NT5E*, *BCL11A and ANGPT1)* were upregulated and the rest 9 KGs were downregulated (**Table 2**). Further information of 13 kg is included in (**S2 Table in S2 File**).

**Table 2:** Selection of KGs by taking the union of five-sets of top-ranked 8 genes produced by five topological measures with the PPI network.

Degree (D)	BottleNeck (E)	Betweenness(F)	Stress (G)	Clustering Coefficient (H)	Key genes (*D*∪*E*∪*F*∪*G*∪*H*)
*STAT1*	*FN1*	*EGR1*	*EGR1*	*SLCO2A1*	***OAS1*, *FN1*, *SLCO2A1*, *HPGD*, *IRF9*, *NQO1*, *AKR1C1*, *OAS3*, *NT5E*, *TP53INP1*, *ANGPT1*, *ATF6B*, *BCL11A***
*EGR1*	*EGR1*	*FN1*	*FN1*	*HPGD*	
*OAS1*	*STAT1*	*STAT1*	*STAT1*	*TIMP3*	
*OAS2*	*REC8*	*REC8*	*REC8*	*NQO1*	
*IRF9*	*TRIP13*	*TRIP13*	*TRIP13*	*AKR1C1*	
*OAS3*	*NT5E*	*NT5E*	*NR2F1*	*CENPN*	
*RSAD2*	*NR2F1*	*NR2F1*	*ATF6B*	*TP53INP1*	
*IFIT3*	*RELB*	*RELB*	*BCL11A*	*ANGPT1*	

### GO terms and KEGG pathway enrichment analysis of DEGs highlighting KGs

The GO functional enrichment analysis of DEGs showed that 46 GO-BP terms, 11 GO-CC terms and 14 GO-MF terms are enriched by the downregulated genes, where KGs were involved with 25 BPs, 8 CCs and 10 MFs. On the other hand, 15 BPs, 3 CCs and 4 MFs are enriched by the upregulated genes, where KGs were directly involved with 5 BPs (**S3 Table in S2 File**). Among the enriched GO functions including downregulated KGs, 5 GO-BP terms (GO:0060337~type I interferon signaling pathway, GO:0051607~defense response to virus, GO:0060333~interferon-gamma-mediated signaling pathway and GO:0055114~oxidation-reduction process), 2 GO-CC terms (GO:0005737~cytoplasm and GO:0005578~proteinaceous extracellular matrix), and 1 GO-MF terms (GO:0016491~oxidoreductase activity) were reported by other researchers that association with BC (see **Table 3** and discussion section for more details). The upregulated KGs involving 2 GO-BP terms (GO:0045944~positive regulation of transcription from RNA polymerase II promoter and GO:0000122~negative regulation of transcription from RNA polymerase II promoter), 1 GO-CC terms (GO:0072559~NLRP3 inflammasome complex) and 1 GO-MF terms (GO:0097153~cysteine-type endopeptidase activity involved in apoptotic process) were also reported by other researchers that association with BC (see **Table 3** and discussion section for more details as before). The KEGG pathway enrichment analysis of DEGs showed that 8 and 2 pathways are enriched by the downregulated and upregulated KGs, respectively. Among them, downregulated KGs involving hsa05168:Herpes simplex infection pathway and upregulated KGs involving hsa04151:PI3K-Akt signaling pathway were also reported by other researchers as the pathways of BC development (**Table 3**).

**Table 3:** Significantly enriched GO functions and KEGG pathways by the DEGs involving KGs that were also supported by the literature review about their association with BC and other cancers.

GO Terms/Functions	DEGs (Counts)	*P*-Value	Associated KGs
Downregulated DEGs			
GO Terms of Biological Processes (BPs)			
GO:0060337~type I interferon signaling pathway [46]	12	8.41E-13	*OAS1*, *OAS3*, *IRF9*
GO:0051607~defense response to virus [47]	12	2.72E-08	*OAS1*, *OAS3*, *IRF9*
GO:0060333~interferon-gamma-mediated signaling pathway [47]	6	1.53E-04	*OAS1*,*OAS3*, *IRF9*, *ATF6B*
GO:0045071~negative regulation of viral genome replication [48]	5	1.86E-04	*OAS1*, *OAS3*
GO:0055114~oxidation-reduction process [49]	9	0.06191	*NQO1*, *HPGD*, *AKR1C1*
GO Terms of Cellular Components (CCs)			
GO:0005737~cytoplasm [50]	57	4.91E-05	*OAS1*, *OAS3*, *IRF9*, *HPGD*, *TP53INP1*, *NQO1*
GO:0005578~proteinaceous extracellular matrix [8]	7	0.009721	*FN1*
GO Terms of Molecular Function (MF)			
GO:0016491~oxidoreductase activity [51]	5	0.032918	*HPGD*, *AKR1C1*
Upregulated DEGs			
GO Terms of BP			
GO:0045944~positive regulation of transcription from RNA polymerase II promoter [52]	7	0.014821	*BCL11A*, *SLCO2A1*
GO:0000122~negative regulation of transcription from RNA polymerase II promoter [53]	6	0.015973	*BCL11A*, *NT5E*
GO Terms of CC			
GO:0072559~NLRP3 inflammasome complex [54]	2	0.017428	*FN1*
GO Terms of MF			
GO:0097153~cysteine-type endopeptidase activity involved in apoptotic process [55]	2	0.02663	*FN1*
KEGG pathways	DEGs (Counts)	*P*-Value	Associated KGs
Downregulated DEGs			
hsa05168:Herpes simplex infection [56]	6	0.012895	*OAS1*, *OAS3*, *ATF6B*, *IRF9*
Upregulated DEGs			
hsa04151:PI3K-Akt signaling pathway [57]	4	0.030138	*ANGPT1*, *SLCO2A1*, *BCL11A*

### Regulatory network analysis of KGs

**Fig 4:**
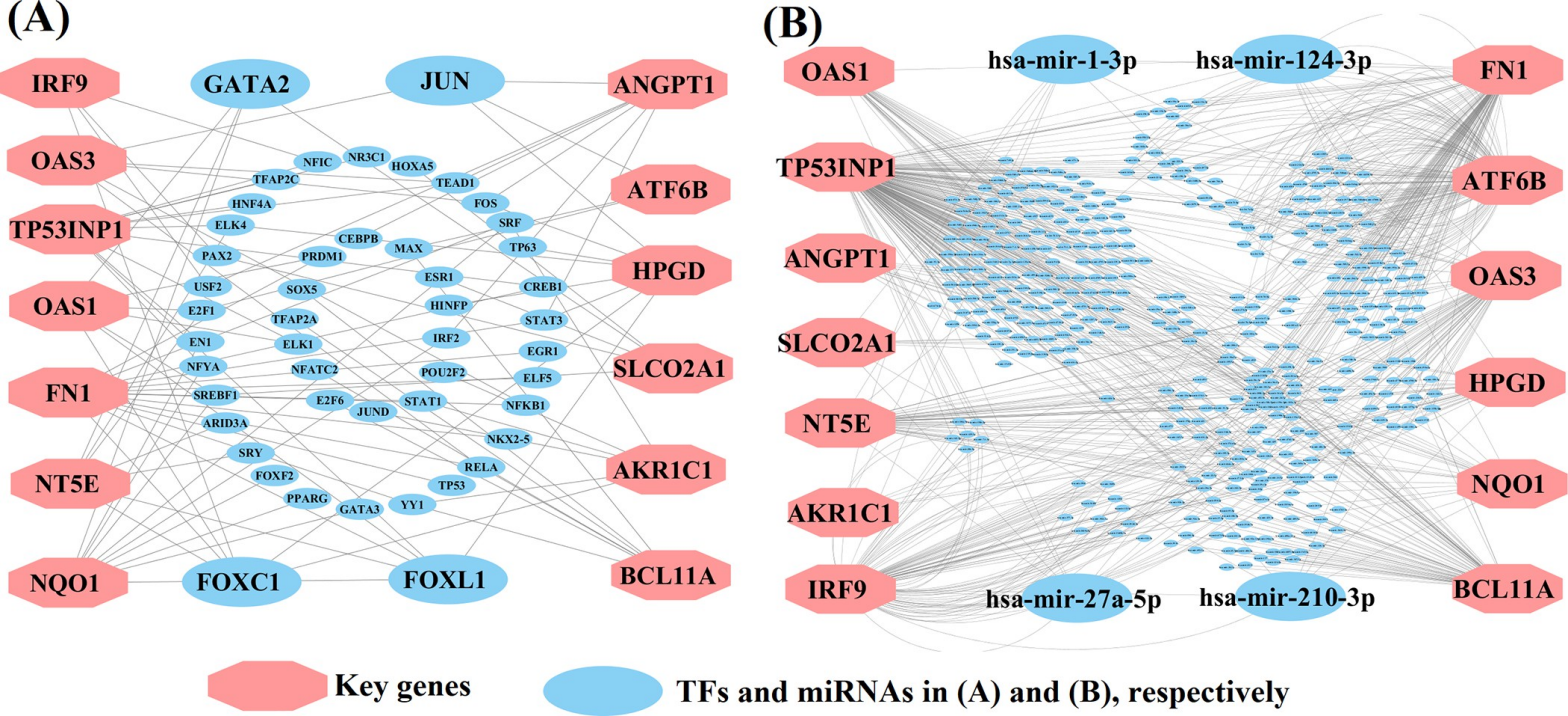
KGs regulatory network analysis results (**A**) KGs-TFs interaction network to identify key transcriptional regulators of KGs, (**B**) KGs-miRNAs interaction network to identify key post-transcriptional regulators of KGs. Here pink color octagon indicates the KGs in both **A** and **B**, blue color bigger size ellipse indicates key TFs in **A** and key miRNAs in **B**.

We constructed KGs versus transcription factors (KGs-TFs) interaction network to identify top ranking TFs as the key transcriptional regulators of KGs. We selected top 4 key TFs (FOXC1, FOXL1, JUN, and GATA2) as the vital transcriptional regulators of KGs with degree > 4, where large blue ellipses indicate top degree key TFs and pink octagons indicate KGs in **Fig 4A**. To identify top ranking micro-RNA (miRNA) as the key post-transcriptional regulators of KGs, we constructed KGs-miRNAs interaction network. We selected top 4 key miRNAs (hsa-miR-27a-5p, hsa-miR-124-3p, hsa-miR-1-3p, and hsa-miR-210-3p) as the vital regulators of KGs with degree > 7, where large blue ellipses indicate top degree key miRNAs and pink octagons indicate KGs in **Fig 4B**.

### Prognostic power analysis

**Fig 5:**
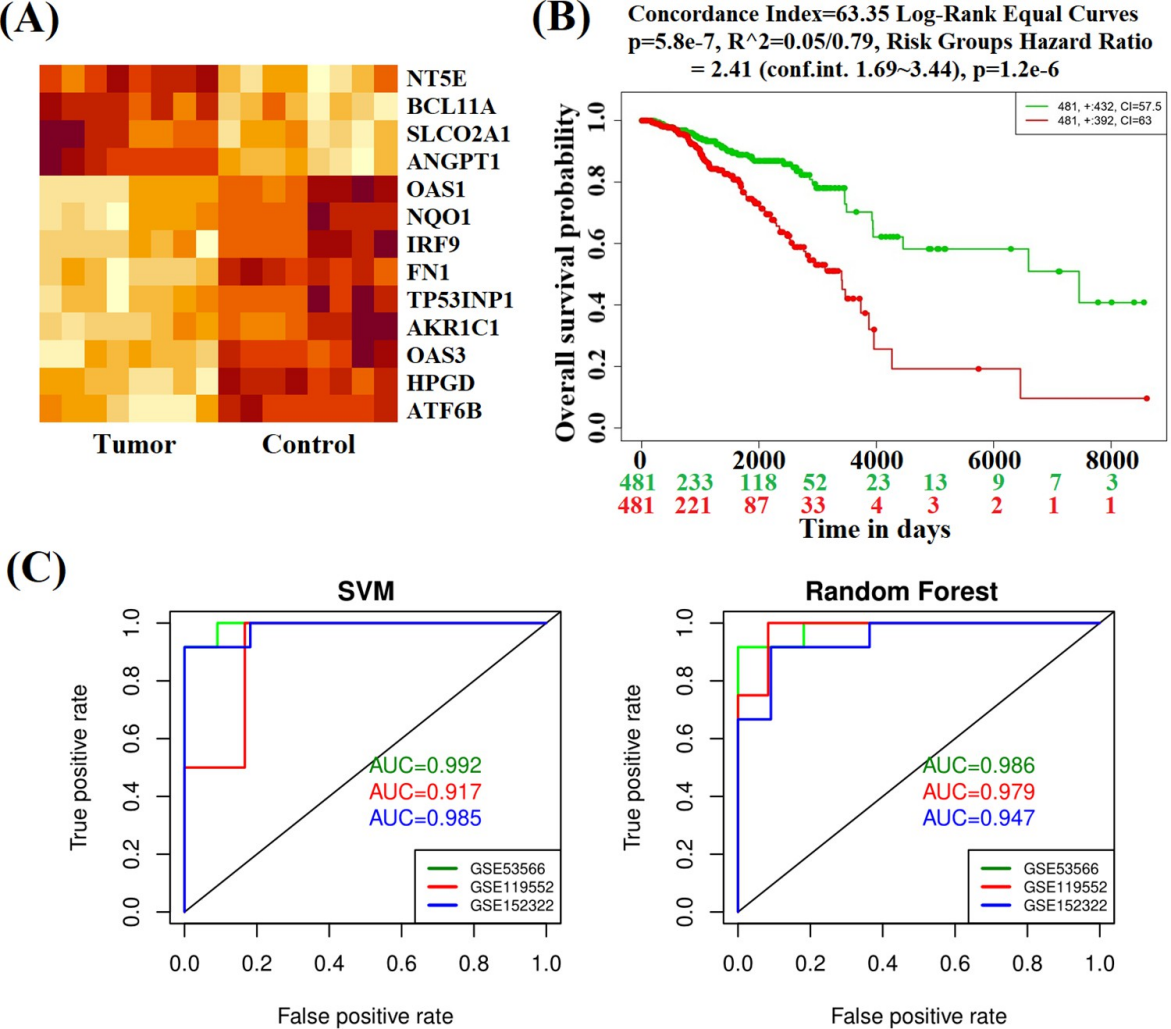
The prognostic powers of KGs were displayed by **(A)** Heatmap of hierarchical clustering **(B)** Multivariate survival curves with KGs and **(C)** ROC curves of prediction models with KGs.

We considered both supervised and unsupervised learning’s including multivariate survival analysis to investigate the prognostic power of 13 KGs (**Fig 5**). The **Fig 5A** shows that KGs are able to classify case and control samples accurately by the unsupervised hierarchical clustering (HC). The multivariate survival curves based on the expressions of 13 KGs, separated the low (control) and high (BC) risk groups significantly (see **Fig 5B**). In the case of supervised learning, we trained two popular classifiers (RF and SVM) by taking the expression profiles of 13 KGs from all samples (8 BC and 8 controls) of the study dataset with the NCBI accession number GSE53566. Then we investigated their prediction performance using both training and independent test datasets. We investigated the training performance by taking all samples (8 BC and 8 controls). To investigate their test performance unbiasedly, we considered the expression profiles of 13 KGs from two independent GEO datasets with the NCBI accession numbers GSE119552 and GSE152322, respectively. The dataset GSE119552 consisted of 12 tumors and 4 control samples, the other test dataset GSE152322 consisted of 11 tumors and 12 control samples. We classified all samples from each dataset by the prediction models. **Fig 5C** showed the training performance (green color) and independent test performance (blue and red color). We observed that both training and independent test performance are good and reasonable (AUC>0.90) for each of training and independent test datasets.

### Exploring candidate drugs by molecular docking analysis

**Fig 6:**
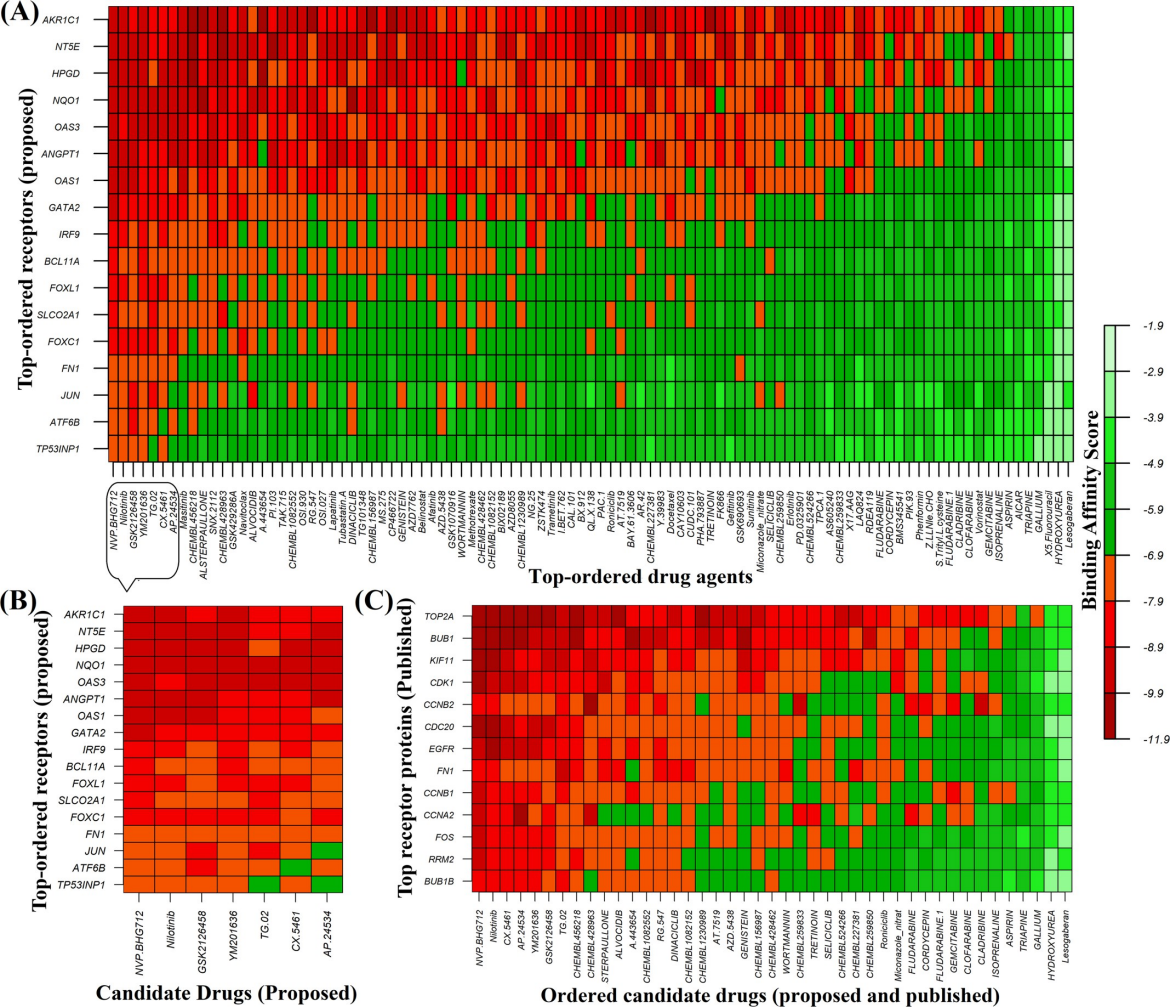
Molecular docking simulation results for exploring candidate drugs against BC. **(A)** Image of binding affinity scores of proposed ordered receptor proteins with the top 97 ordered meta-drug agents, **(B)** Image of binding affinity scores of proposed ordered receptor proteins with the proposed ordered candidate drugs only **(C)** Image of binding affinity scores of ordered proposed and already published candidate drugs against the top-ranked independent receptors published by others.

**Fig 7:**
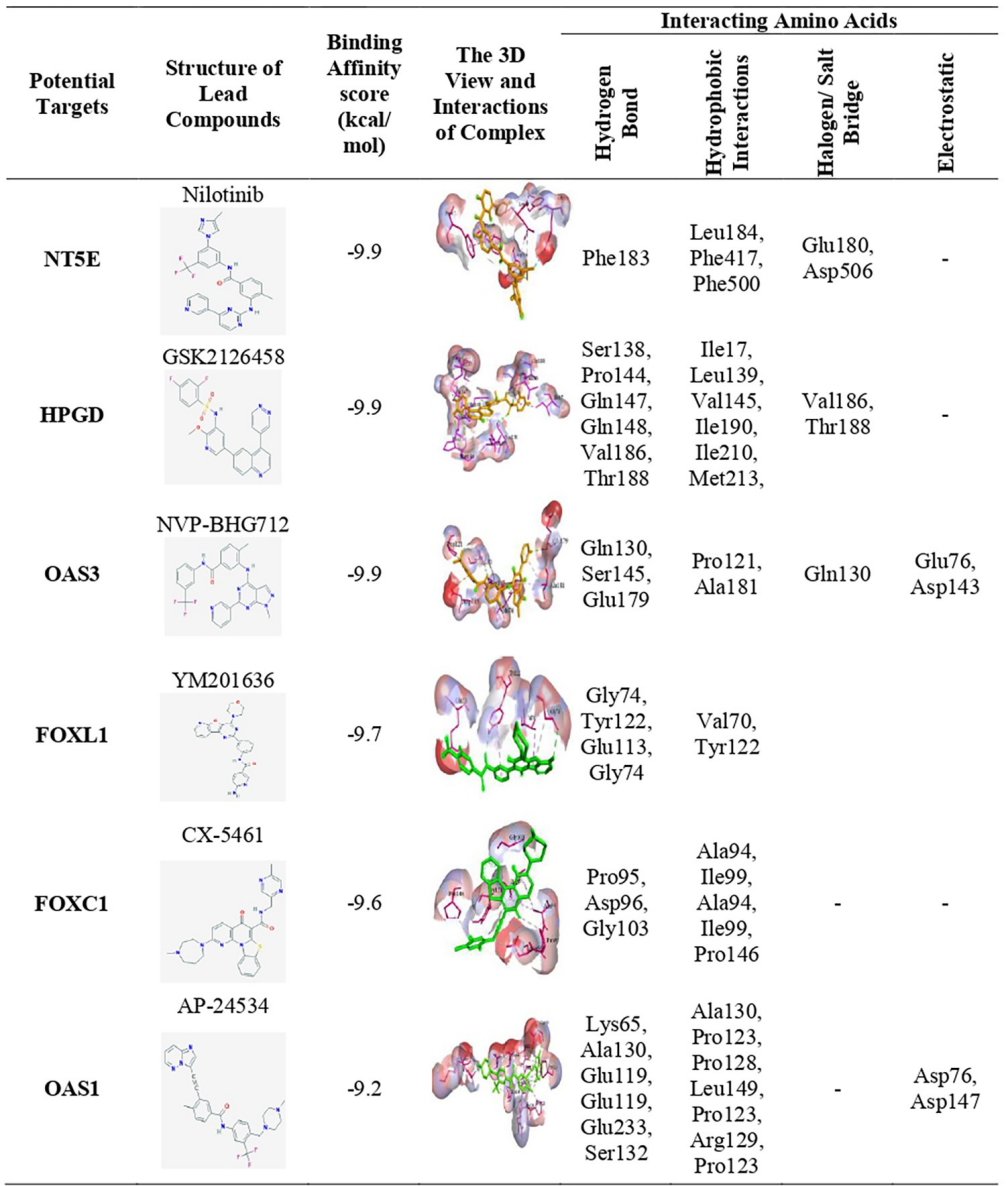
The 3D views of the selected strong binding interactions between drug targets and agents were displayed. The key interacting amino acids and their binding types with potential targets were also shown.

To explore candidate drugs for breast cancer (BC), we considered 13 KGs based proteins (*AKR1C1*, *IRF9*, *OAS1*, *OAS3*, *SLCO2A1*, *NT5E*, *NQO1*, *ANGPT1*, *FN1*, *ATF6B*, *HPGD*, *BCL11A*, and *TP53INP1*) and its regulatory key 4 TFs proteins (FOXC1, GATA2, FOXL1 and JUN) as the *m* = 17 drug target receptors. The 3-Dimension (3D) structure of NT5E, HPGD, NQO1, OAS1, ANGPT1, IRF9, BCL11A, SLCO2A1, FN1, OAS3, AKR1C1, JUN and GATA2 were downloaded from Protein Data Bank (PDB) with the PDB codes 6S7F, 2GDZ, 5FU, 4RWP, 4JYO, 5OEN, 6KI6, 3MRR, 2HAZ, 4S3N, 3C3U, 1A02 and 5O9B and rest of them such as ATF6B, TP53INP1, FOXC1, and FOXL1 targets were downloaded from SWISS-MODEL using UniProt with IDs Q99941, Q96A56, Q12948 and Q12952 respectively. Then we considered 82 meta-drug molecules from the GSCALite database and 47 meta-drugs from the published articles as drug agents (see S1 File (Tables 2 and 3)). The 3D structures of drug agents were downloaded from the PubChem database. Then we performed molecular docking analysis between our proposed receptors and meta-drug agents. The binding affinity score matrix between the ordered receptors and ordered drug-agents were displayed in **Fig 6A**. We observed that top order four lead compounds/drugs (NVP-BHG712, Nilotinib, GSK2126458, and YM201636) produce highly significant binding affinity scores with all m = 17 target proteins, and their average binding affinity scores across all receptors were -8.65, -8.55, -8.50, and -8.45 (kcal/mol), respectively. The next two top ordered drugs (TG-02 and CX-5461) produced highly significant binding affinity scores with 16 target proteins, and their average binding affinity scores across all m = 17 targets were -8.40 and -8.22, respectively. The 7^th^ top ordered drug AP-24534 produced significant binding affinity scores with 14 target proteins and the average binding affinity score was -8.0. The other drugs (lead compounds) produced significant binding affinity scores with less than 12 target proteins out of 17 and their average binding affinity scores were negatively smaller then -7.5. Therefore, we considered top ordered seven drugs (NVP-BHG712, Nilotinib, GSK2126458, YM201636, TG-02, CX-5461 and AP-24534) as the candidate drugs in our study and highlighted them in **Fig 6B**. We also examined their complete interaction profile including hydrogen bonds, hydrophobic, halogen/ salt Bridge and electrostatic interactions in **Fig 7**.

### Performance investigation of proposed drugs by cross-validation

To investigate the resistance performance of the proposed drugs against the state-of-the-art alternative receptors for BC compared to the transcriptome-guided 47 published drugs, we performed molecular docking analysis of our proposed drugs including all published drugs with the top ranked independent receptors (KIF11, RRM2, BUB1, CDC20, FOS, FN1, BUB1B, CCNB2, CCNA2, CDK1, TOP2A, CCNB1, and EGFR) published by others for BC in different 78 articles (see S1 File (Table 3)). The 3D structure of KIF11, RRM2, BUB1, CDC20, FOS, FN1, BUB1B, CCNA2, CDK1, TOP2A, CCNB1, and EGFR were downloaded from PDB database with the PDB codes 1Q0B, 3BS9, 4a1g, 1DUJ, 1FXL, 2HAZ, 2WVI, 1VIN, 6GU6, 1ZXM, 2B9R, and 3G5Z respectively and for another one CCNB2 downloaded from SWISS-MODEL using UniProt with ID O95067. The **Fig 6C** showed the resistance performance of our proposed drugs in a comparison of the publicly available drugs against the top ranked 13 independent receptors. We observed that our proposed drugs showed better performance compare to the published drugs in terms of negatively highest binding affinities with the independent receptors. Therefore, we can strongly recommend that the proposed drugs might be more effective candidate than the published drugs for the treatment against BC.

## Discussion

**Fig 8:**
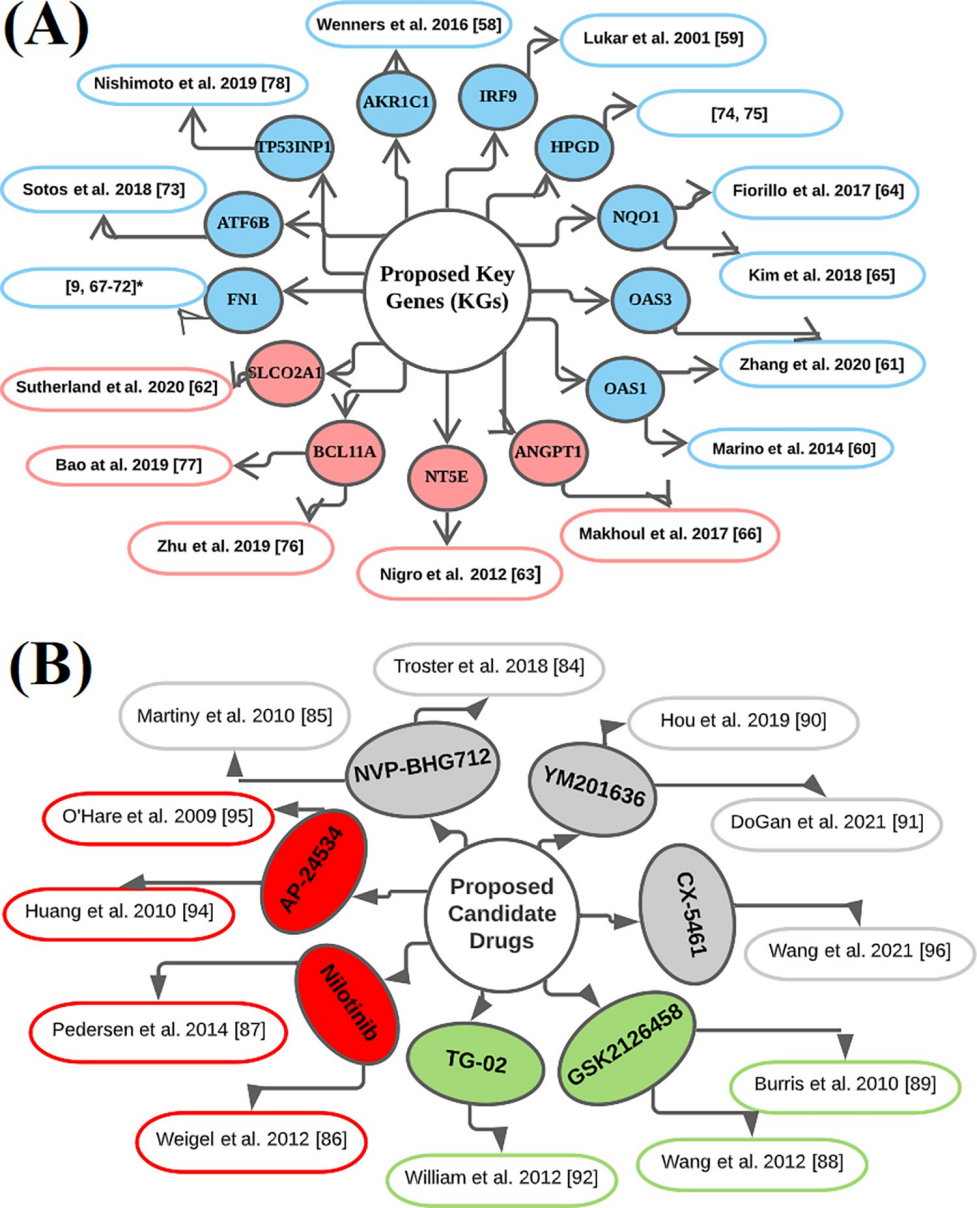
Validation of the proposed KGs (receptors) and candidate drugs in favor of BC by the literature review **(A)** Validation of the proposed KGs: circles with blue color indicate downregulated KGs and pink color indicates upregulated KGs, and each connected network with a circle indicates the reference in which the KG is associated with BC, **(B)** Validation of the proposed candidate drugs: circles with red color indicate FDA approved and investigational drugs, green color indicate investigational drugs and ash color indicate unapproved drugs, and each connected network with a circle indicates the references in which our suggested drugs might be effective against BC treatment.

In this study, we identified key genomic biomarkers highlighting their pathogenetic processes for breast cancer (BC) diagnosis, prognosis and therapies. At first, we identified 190 DEGs (138 downregulated and 52 upregulated) from the publicly available microarray gene-expression profiles. Then we detected 13 DEGs (*AKR1C1*, *IRF9*, *OAS1*, *OAS3*, *SLCO2A1*, *NT5E*, *NQO1*, *ANGPT1*, *FN1*, *ATF6B*, *HPGD*, *BCL11A*, and *TP53INP1*) as the KGs that drive the progression of BC. Some literatures also suggested that these KGs are BC causing genes [9, 58–78] (see **Fig 8A**). For example, the expression of two genes (AKR1C1 and AKR1C2) in carcinoma cells and stromal fibroblasts and their positive correlation are favorable tumor characteristics in primary BC patients [58]. Also these two genes appear to be an interesting target for new hormone-based therapy strategies in primary BC. The *IRF9* gene with overexpression has the potential to be a surrogate marker of response and may be associated with drug resistance for BC [59]. The expression of the OAS1 gene that was inversely associated with multiple MSGs in the BC cell line [60]. The OAS3 gene plays a prognostic role in BC patients with potential mechanical value [61]. The SLCO2A1 is ubiquitously expressed and marked as a prostaglandin transporter due to its high affinity [62]. The Gene NT5E is regulated epigenetically in BC, the epigenetic status of this gene influencing metastasis and clinical outcome, and suggests that NT5E CpG island methylation is a promising BC epigenetic biomarker [63]. The Pharmacological prohibition of *NQO1* and *GCLC* is a new therapeutic strategy for overcoming tamoxifen-resistance and also shows that the prediction of *NQO1* as a biomarker has the significant prognostic value of tumor recurrence in BC patients [64]. β-lapachone (bL) may be a therapeutic targeting for BC stem-cells with appropriate *NQO1* expression [65]. Germline Genetic Variants in *ANGPT1*, *ANGPT2*, *TEK*, *MMP9*, *VEGFA* and *FGF2* are involved with Pathologic complete reaction to Bevacizumab in BC Patients [66]. Several studies have suggested that the gene FN1 is highly associated with BC [9, 67–72]. They suggested that genetic variants of the ATF6B gene were associated with modified relationships between reproductive factors and BC [73]. A new BC risk variant rs8752 in HPGD in Chinese women’s through a systematic case-control study of microRNA binding site SNPs [74, 75]. The key gene BCL11A plays a crucial role in BC tumorigenicity and stemness maintenance through activating Wnt/β-catenin signaling pathway, and may become a potential target for the treatment of BC [76]. Moreover, another study identified 4 hub genes (*BCL11A*, *FOXC1*, *RGMA*, and *FAM171A1*) that showed a highly positive correlation with the triple-negative BC subtype [77]. Low expression of *TP53INP1* is an independent factor of poor prognosis in BC patients, especially ERα-positive patients and may become a potential therapeutic target in ERα-positive BC patients [78].

The GO functional and KEGG pathway enrichment analyses of DEGs significantly revealed some GO terms of BPs, MFs and CCs, and KEGG pathways by involving KGs that are highly linked with BC patients (see **Table 3**). Our literature review also supported their link with BC. As for examples with the enriched BPs, the cell growth inhibition of MCF-7 (the BC cell line) significantly increases after treatment of BC with XN by influencing the *type I interferon signaling pathway* [46] that is associated with three KGs (*OAS1*, *OAS3* and *IRF9*). Two GO terms *defense response to virus* (associated with 3 KGs: OAS1, OAS3 and IRF9) and *interferon-gamma-mediated signaling pathway* (associated with 4 KGs: OAS1, OAS3, IRF9 and ATF6B) were reported as two important BPs for BC progression [47]. Two KGs (*OAS1*, *OAS3)* involving n*egative regulation of viral genome replication* process influence the BC enriched dysregulated subnetworks and play a potential role in cardiotoxicity [48]. The *oxidation-reduction process* associated 3 KGs (*NQO1*, *HPGD* and *AKR1C1*) is functionally enriched with multiple cancer type-specific metastasis over-expression signatures [49]. Biological process analysis has shown that *positive regulation of transcription from RNA polymerase II promoter* (associated with *BCL11A* and *SLCO2A1*) and *negative regulation of transcription from RNA polymerase II promoter* (associated *BCL11A* and *NT5E*) are functionally enriched for DEGs of BC [52, 53]. Among the enriched CCs, The *cytoplasm* (associated with 6 KGs: *OAS1*, *OAS3*, *IRF9*, *HPGD*, *TP53INP1* and *NQO1*) has been found to be associated with most proteins that are highly expressed for cancer [50]. Upregulated DEGs for BC are functionally enriched in the *proteinaceous extracellular matrix* (associated KGs: *FN1*) pathway [79]. It was suggested that activation of the *NLRP3 inflammasome complex* (associated KGs: *FN1*) would be an innovative therapeutic pathway to control tumor growth [54]. Inhibition of NADH: ubiquinone *oxidoreductase activity (*associated KGs: *HPGD* and *AKR1C1)* blocks multiple signal transduction pathways in MCF-7 human BC cells through rotenoids drug [51]. The 320 differential expressed microRNAs targeted genes for BC were functionally enriched in negative regulation of *cysteine-type endopeptidase activity involved in apoptotic process* (Associated KGs: *FN1*) [55]. After treatment of a metastatic BC patient with *herpes simplex infection pathway (associated KGs*: *OAS1*, *OAS3*, *ATF6B*, *IRF9)*, there has created a case of Sweet Syndrome[56]. Among the enriched KEGG pathways, Paclitaxel inhibits the proliferation and invasion of the MCF-7 cell in *PI3K-AKT signaling pathway* (Associated KGs: *ANGPT1*, *SLCO2A1 and BCL11A*) to prevent BC [57].

The KGs-TFs interaction network analysis indicated that 4 TFs proteins (FOXC1, FOXL1, GATA2, and JUN) are the key transcriptional regulatory factors of Kgs (see **Fig 4A**). Among them FOXC1 (a regulator of *NT5E*, *IRF9*, *AKR1C1*, *HPGD*, *FN1* and *OAS1*) is connected with lymphatic vessel formation, arterial cell specification, and cardiovascular development [80]. The expression of TF-protein FOXL1 (a regulator of *NT5E*, *TP53INP1*, *HPGD*, *FN1*, *OAS1* and *NQO1*) is connected with numerous cancer [81]. The TF-protein GATA2 (a regulator of *NT5E*, *SLCO2A1*, *OAS1* and *NQO1*) is connected with Hematopoietic and immune defects [82]. The TF-protein JUN (a regulator of *ANGPT1*, *HPGD*, *ATF6B* and *OAS3*) is associated with bladder cancer disease [83]. We also constructed the proteins-disease interaction network to detect other diseases that are also connected with the proposed KGs. Total 8 KGs out of 11 were associated with others 156 diseases that can be considered as the non-causal risk factors of BC. Especially, two diseases "Autosomal recessive predisposition" and "Schizophrenia" were mostly related with our target proteins.

To investigate the prognostic power of KGs, we performed multivariate survival analysis and developed two prediction models through two classifiers (SVM and RF) in **Fig 5**. Our developed two prediction models showed good performance with both training and test datasets generated from the main data collected from NCBI with accession number GSE53566. The AUC values were 0.992 and 0.986 for SVM and RF based models for the training dataset, respectively. To investigate their performance unbiasedly, we also considered two independent test datasets from other NCBI sources with accession numbers GSE119552 and GSE152322, respectively. We observed that both predictors show good performance with both independent test datasets. The values of AUC were 0.917 and 0.979 for independent test dataset-1 and 0.985 and 0.947 for the independent test dataset-2 based on SVM and RF models, respectively. These results indicate the good prediction performance for the identified KGs, so we suggested the prognostic model for the two classifiers (SVM and RF).

To explore our proposed KGs-guided new and repurposable candidate drugs for the treatment against BC, we considered the proposed KGs based 13 key proteins (AKR1C1, IRF9, OAS1, OAS3, SLCO2A1, NT5E, NQO1, ANGPT1, FN1, ATF6B, HPGD, BCL11A, and TP53INP1) and their regulatory 4 TFs proteins (FOXC1, FOXL1, GATA2, and JUN) as the drug target receptors and performed their docking simulation with 129 drug molecules collected from the GSCALite database and published articles (see **Fig 6A**). Then we selected top-ranked 7 drugs (NVP-BHG712, Nilotinib, GSK2126458, YM201636, TG-02, CX-5461, and AP-24534) as the most probable repurposable candidate drugs for BC patients based on their strong binding affinity scores (less than -7.0 kcal/mol) with all the target proteins (see **Fig 6A and 6B**). Then we investigated the resistance performance of both the proposed and already published candidate drugs against the state-of-the-art alternatives top-ranked 13 independent receptors suggested by others for BC and observed that our proposed candidate drugs are more effective compared to the already published drugs against the independent receptors also (see **Fig 6C**). We also validated our proposed drugs in favor of BC by the literature review (**see Fig 8B**).

Among the identified candidate drugs NVP-BHG712 had the knack to inhibit EphB4 kinase activity and EphA2 with an IC_50_ of 3 nM in HEK293 T cells. Besides, NVP-BHG712 had a good binding score for other Eph targets as well, with IC50s ranging from 0.3 nM to 303 nM for EphA3 and EphA1 respectively. Overall the isomers had a low binding score with IC_50_ ranging from 163 to 1660 nM for EphA2 and EphB4, respectively, which revealed that small changes could be made a significant effect on Eph target binding [84]. NVP-BHG712 had the ability to inhibit VEGFR2 as well, but the compound has a 200 times higher binding score for EphB4 [85]. Preclinical studies showed that nilotinib had a growth inhibitory effect on LTED (long-term estrogen deprived) MCF-7 BC cells via ER [86]. Also the nilotinib and sorafenib were considered as potential new treatment options for tamoxifen‑resistant BC [87]. GSK2126458 had been considered as potential therapies for BC and were highly selective and effective small compounds inhibitors that receptor both multiple class I PI3K isoforms and mTOR kinase activity [88, 89]. YM201636 was exposed with validate through vivo analysis that YM201636 have an inhibitory effect on tumour cell growth without any side effects for both liver cancer and non-small cell lung cancer [90, 91]. TG-02 (Zotiraciclib) is used as enzyme inhibitor of CDKs to treat cancer disease and also approved as an orphan drug by FDA to treat glioma disease [92, 93]. AP-24534 (Ponatinib) was proposed as an inhibitor of multi-target drugs to treat chronic myeloid leukemia disease and approved by the FDA in December 2012 as a candidate drug [94, 95]. CX-5461 is an inhibited drug for colorectal cancer (CRC) development in Znf545Δ/ΔApcMin/+ mice [96]. Among the proposed seven candidate drugs, Nilotinib and AP-25534 are approved by the FDA in 2007 and 2012 respectively, TG-02 and GSK2126458 are investigational drugs and three other drugs (NVP-BHG712, YM201636 and CX-5461) are not yet approved. The unapproved drugs should be further assessed in molecular level by the wet-lab experiments in prior to clinical investigation in the treatment of BC.

## Conclusion

The main purpose of this study was to identify potential KGs highlighting their function, pathways, and regulatory factors for breast cancer (BC) diagnosis, prognosis and therapies by using the integrated bioinformatics and statistical approaches. We identified BC causing 13 DEGs (*AKR1C1*, *IRF9*, *OAS1*, *OAS3*, *SLCO2A1*, *NT5E*, *NQO1*, *ANGPT1*, *FN1*, *ATF6B*, *HPGD*, *BCL11A*, and *TP53INP1*) as the KGs by using the five topological measures in the PPI networking results. Their association with BC was also reported by several other studies directly or indirectly that we mentioned in the discussion section. We detected four TFs proteins (FOXC1, FOXL1, JUN, and GATA2) and four microRNAs (hsa-miR-27a-5p, hsa-miR-124-3p, hsa-miR-1-3p, and hsa-miR-210-3p) as the key transcriptional and post-transcriptional regulators of KGs. These regulatory factors play the vital role for the regulation of KGs. The GO terms (BPs, MFs and CCs) and KEGG pathway enrichment analysis revealed some vital GO terms from each of BPs, MFs and CCs that are significantly enriched by DEGs including KGs. The enriched GO terms and KEGG pathways were considered as the key pathogenetic processes of BC progression. These findings were also supported by the literature review directly or indirectly. We investigated the prognostic performance of KGs by using multivariate survival analysis including unsupervised hierarchical clustering and supervised classification. In each case, we observed the strong prognostic performance of the proposed KGs. Then we considered the proposed 13 key proteins and their regulatory 4 TFs-proteins as the drug target receptors to explore effective drugs for BC by molecular docking simulation with the 129 meta-drug agents. We detected 7 small molecules (NVP-BHG712, Nilotinib, GSK2126458, YM201636, TG-02, CX-5461, and AP-24534) as the top ranked candidate drugs for the treatment against BC. Then we investigated the resistance performance of both the proposed and already published candidate drugs against the state-of-the-art alternatives already published top-ranked 13 independent receptors for BC and observed that our proposed candidate drugs are computationally more effective against the independent receptors also. Therefore, the proposed candidate drugs might be played the vital role for the treatment against BC.

## Supporting information

S1 File Supplementary information on datasets. (DOCX)

S2 File Supplementary results. (PDF)
