# Supplementary material for: Integrated bioinformatics and statistical approaches to explore molecular biomarkers for breast cancer diagnosis, prognosis and therapies
Source: PLoS One. 2022 May 26;17(5):e0268967. doi: 10.1371/journal.pone.0268967 (PMC9135200; doi:10.1371/journal.pone.0268967)
Supplement: S1 File — (DOCX) [file pone.0268967.s001.docx]

**S1 File: Supplementary information on datasets**

**Table S1:** Descriptions of microarray gene expression data used in this study.

| **Datasets** | **Accession number** | **Array Type/Platform** | **Number of tissue** | | **Contributors** |
| --- | --- | --- | --- | --- | --- |
|  |  |  | **Tumor** | **Control** |  |
| Train Data | GSE53566 | GPL6480 Agilent-014850 Whole Human Genome Microarray 4x44K G4112F (Probe Name version) | 8 | 8 | Martin NH et al. |
| Test Data1 | GSE119552 | GPL16699 Agilent-039494 SurePrint G3 Human GE v2 8x60K Microarray 039381 (Feature Number version) | 12 | 4 | Lecomte S et al. |
| Test Data12 | GSE152322 | GPL10558 Illumina HumanHT-12 V4.0 expression beadchip | 11 | 12 | Cataldo A et al. |

**Table S2:** Drug list download from the online database GSCALite (Consider Set A) and collected from published articles (Consider Set B).

| **Drugs List (Set A)** | **Pubchem/Drug bank ID** | **Drugs List (Set B)** | **Pubchem ID** |
| --- | --- | --- | --- |
| 5-Fluorouracil | 3385 | CLADRIBINE | 20279 |
| Methotrexate | 126941 | GALLIUM | 65430 |
| AZD7762 | 11152667 | NITRATE | 10762 |
| CP466722 | 44551660 | MOTEXAFIN | NA |
| BIX02189 | 135659062 | GADOLINIUM | NA |
| NVP-BHG712 | 16747388 | HYDROXYUREA | 3657 |
| QL-XII-61 | NA | CLOFARABINE | 119182 |
| CX-5461 | [25257557](https://pubchem.ncbi.nlm.nih.gov/compound/25257557) | GEMCITABINE | 60750 |
| PAC-1 | 135421197 | FLUDARABINE | 657237 |
| Navitoclax | DB12340 | PHOSPHATE | NA |
| QL-X-138 | 73707530 | TRIAPINE | 9571836 |
| TL-1-85 | NA | FLUDARABINE | 657237 |
| WZ-1-84 | NA | DINACICLIB | 46926350 |
| YM201636 | 9956222 | ALVOCIDIB | 5287969 |
| BMS345541 | 9813758 | Roniciclib | 45380979 |
| 17-AAG | 66576986 | AT-7519 | 91669601 |
| NG-25 | 155489752 | AZD-5438 | 16747683 |
| SNX-2112 | 24772860 | TG-02 | 16739650 |
| XMD13-2 | NA | CHEMBL1236539 | NA |
| TPCA-1 | 9903786 | RG-547 | 6918852 |
| GSK429286A | 60146047 | SELICICLIB | 160355 |
| Y-39983 | 139292187 | SURAMIN | NA |
| Docetaxel | DB01248 | CORDYCEPIN | 6303 |
| CEP-701 | 126565 | GENISTEIN | 5280961 |
| Masitinib | 10074640 | CHEMBL1235116 | 4722579 |
| QL-XI-92 | NA | ASPIRIN | 2244 |
| TG101348 | 16722836 | Lesogaberan | 9833984 |
| WZ3105 | NA | A-443654 | 10172943 |
| AZD8055 | 25262965 | CHEMBL524266 | 16757525 |
| OSI-027 | DB12387 | CHEMBL428963 | 24798741 |
| TAK-715 | 9952773 | ALSTERPAULLONE | 5005498 |
| I-BET-762 | 46943432 | CHEMBL156987 | 448008 |
| Ispinesib Mesylate | 6450816 | CHEMBL227381 | 5327109 |
| Genentech Cpd 10 | NA | CHEMBL428462 | 11175137 |
| GSK1070916 | 46885626 | CHEMBL259850 | 448014 |
| Gefitinib | 123631 | CHEMBL456218 | 2079916 |
| Lapatinib | 208908 | CHEMBL1082152 | 11560568 |
| PD-0325901 | 10117717 | STAUROSPORINE | 44259 |
| RDEA119 | 44182295 | CHEMBL1082552 | 135911866 |
| Trametinib | 11707110 | CHEMBL259833 | 24798742 |
| ZSTK474 | 11647372 | CHEMBL1230989 | 33113 |
| KIN001-244 | NA | ARSENIC | NA |
| PI-103 | 83485 | TRIOXIDE | NA |
| GSK690693 | 16725726 | TRETINOIN | 444795 |
| KIN001-102 | NA | WORTMANNIN | 312145 |
| BX-912 | 11754511 | SF-1126 | NA |
| PIK-93 | NA | ISOPRENALINE | 3779 |
| UNC0638 | NA |  |  |
| BAY 61-3606 | 11784504 |  |  |
| AT-7519 | 91669601 |  |  |
| THZ-2-49 | NA |  |  |
| PHA-793887 | 72941850 |  |  |
| Afatinib | 10184653 |  |  |
| Cetuximab | NA |  |  |
| Erlotinib | 176870 |  |  |
| MS-275 | 4261 |  |  |
| CUDC-101 | 24756910 |  |  |
| Belinostat | 6918638 |  |  |
| NPK76-II-72-1 | NA |  |  |
| CAY10603 | 24951314 |  |  |
| AR-42 | 6918848 |  |  |
| Tubastatin A | 49850262 |  |  |
| Vorinostat | 5311 |  |  |
| AICAR | 17513 |  |  |
| KIN001-270 | NA |  |  |
| Phenformin | 8249 |  |  |
| FK866 | plm |  |  |
| Nilotinib | 644241 |  |  |
| AP-24534 | 24826799 |  |  |
| Z-LLNle-CHO | 11754711 |  |  |
| TL-2-105 | NA |  |  |
| OSI-930 | DB05913 |  |  |
| JW-7-24-1 | NA |  |  |
| KIN001-260 | NA |  |  |
| Sunitinib | 5329102 |  |  |
| THZ-2-102-1 | NA |  |  |
| S-Trityl-L-cysteine | 76044 |  |  |
| AS605240 | 5289247 |  |  |
| CAL-101 | 66576979 |  |  |
| GSK2126458 | 25167777 |  |  |
| LAQ824 | 6445533 |  |  |

**Table S3:** List of 77 review articles with their identified hub genes and drugs.

| **Reference &Year** | **Hub-Genes** | **Drugs** |
| --- | --- | --- |
| Chand et al. 2012 [1] | CHUK, INSR and CREBBP | NA |
| Chen et al. 2020 [2] | HSP90AA1, SRC, HSPA8, ESR1, ACTB, PPP2CA, and RPL4. | NA |
| Chuan et al. 2020 [3] | CXCR4 and CXCL10 | NA |
| Dashti et al.2020 [4] | MAD2L1, CCNA2, RAD51-AS1 and LINC01089 | NA |
| Dong et al. 2018 [5] | EGFR, JUN, IGF1, and ESR1 | NA |
| Hao et al. 2020 [6] | RRM2, CDC20, CCNB2, BUB1B, CDK1, and CCNA2 | YES, Index1 |
| Wenners et al. 2016 [7] | AKR1C1 and AKR1C2 | NA |
| Luker et al. 2001 [8] | IRF9 | NA |
| Marino et al. 2014 [9] | PDE5A, UGT1A, IL11RA, DNM3 and OAS1 | NA |
| Zhang et al. 2020 [10] | OAS1, OAS2, OAS3 and OASL | NA |
| Sutherland et al. 2020 [11] | SLCO2A1, SLCO3A1, SLCO4A1 and SLCO5A1 | NA |
| Nigro et al. 2012 [12] | NT5E | NA |
| Fiorillo et al. 2017 [13] | NQO1 and GCLC | NA |
| Kim et al. 2018 [14] | NQO1 | NA |
| Makhoul et al. 2017 [15] | ANGPT1, ANGPT2, TEK, MMP9, VEGFA and *FGF2* | NA |
| Yuan et al. 2019 [16] | FN1, EGFR, JAK3, TUBB3 and PTPRC | NA |
| Yan et al. 2021 [17] | FN1, DDR2, NRP1, EFNA3 and RHOQ. | NA |
| Amjad et al. 2020 [18] | SMC4, FN1, FOS, JUN, and KIF11 and RACGAP1 | NA |
| Wang et al. 2018 [19] | JUN, FOS, ATF3, STAT1, COL1A1 and FN1 | NA |
| Liu et al. 2015 [20] | FN1, IL6 and FOS | NA |
| Ming et al. 2017 [21] | FN1, CD44, NGF, SERPINE1 and CCNA2 | NA |
| Yang et al. 2017 [22] | FN1 | NA |
| Sotos et al. 2018 [23] | RPS6KA1, ATF6B, HSD17B3, CREB3L1 and SRC | NA |
| He et al. 2014 [24] | HPGD | NA |
| Bhar et al.2013 [25] | CCL2, CD47, NFIB, BRD4, HPGD, CSNK1E, NPC1L1, PTEN, PTPN2 and ADAM9 | NA |
| Zhu et al. 2019 [26] | BCL11A | NA |
| Bao et al. 2019 [27] | FOXC1, BCL11A, FAM171A1 and RGMA | NA |
| Nishimoto et al. 2019 [28] | TP53INP1 | NA |
| Li et al. 2018 [29] | AURKA, BIRC5, BUB1B, BUB1, CCNB1, CDK1, KIF11, MAD2L1, NDC80, and PLK1 | NA |
| Xuemei et al. 2019 [30] | TMEM252, PRB2, SMCO1, IVL, SMR3B and COL9A3 | NA |
| Lu et al. 2019 [31] | PHLPP1, UBC, ACACB, TGFB1, and ACTB | NA |
| Li et al. 2020 [32] | CCNE1, EXO1 and TTK | NA |
| Yang et al. 2019 [33] | CCNB2, BUB1, NDC80, CENPE, KIF2C, TOP2A, MELK, TPX2, CKS2 and KIF20A | NA |
| PENG et al. 2017 [34] | P53, GAPDH, CCND1, HRAS and PCNA | NA |
| WANG et al. 2018 [35] | NOTCH1 and MAPK14 | NA |
| JIN et al. 2019 [36] | AURKA, BIRC5, BUB1B, CCNB1, CCNB2, CDC20, CDK1, CDKN3, CENPF, PRC1, PTTG1, TOP2A, TPX2, UBE2C and ZWINT | NA |
| Lin et al. 2020 [37] | CCNB1, RAC1, TOP2A, KIF20A, RRM2, ASPM, NUSAP1, BIRC5, BUB1B, and CEP55 | NA |
| Lina et al. 2019 [38] | TOP2A, MAD2L1, FEN1, EPRS, EXO1, MCM4, PTTG1, RRM2, PSMD14, CDKN3, H2AFZ, CCNE2, EGFR, FGF2, BCL2, PIK3R1 | NA |
| Wang et al.2020 [39] | *TOP2B*, *ACLY*, *EFTUD2*, *DDX5*, *DHX9*, *CCT2*, *SRSF1* | NA |
| Yin et al. 2020 [40] | GRM4, SSTR2, PARD6B, PRR15, and COX6C | NA |
| Zhang et al. 2020 [41] | TXN, HSPD1, HSPH1, and ATIC | NA |
| Zhu et al. 2020 [42] | FOS, CCL2, and XCL12 | NA |
| Zhang et al. 2019 [43] | CREB1, ARF3, UBA5, SIAH1, KLHL3, HECTD1, MMP9, TRIM69, MEX3C, ASB6, UBE2Q2, FBXO22, EIF4A3, and PXN. | NA |
| Lv et al. 2020 [44] | PTPRC, IL6, SELL, CD40, and SPN | NA |
| ZHOU et al. 2020 [45] | CXCL8, IL1β and PTGS2. | NA |
| YANG et al. 2018 [46] | NOS3 and BDNF | NA |
| YUZHI et al. 2018 [47] | TOP2A, BIRC5, CDK1, CCNB1, NDC80; | NA |
| Zhou et al. 2020 [48] | CXCL8, CD44, MMP9, and BMP7 | NA |
| Wang et al. 2019 [49] | SIDT1, ANKRD30A, GPR160, and CA12 | NA |
| Bao et al. 2020 [50] | AURKB, GINS2, MCM10, UHRF1, POLE2, SPC24, and E2F2 | NA |
| Zeng et al. 2018 [51] | GSK3B, RAC1, PXN, ERBB2, HSP90AA1, FGF2, PIK3R1 and RAC2 | NA |
| Liu et al. 2021 [52] | FERMT2, ITGA5, ITGB1, MCAM, CEMIP, HGF, TGFBR1, F2RL2 | NA |
| Zhao et al. 2019 [53] | VEGFA, KRAS, CDH2, ZEB2, TWIST1, and NTRK2 | NA |
| Fei et al. 2020 [54] | NAT1, GATA3 and SCUBE2 | NA |
| Alam et al. 2022 [55] | BUB1, ASPM, TTK, CCNA2, CENPF, RFC4, and CCNB1 | Trametinib, selumetinib, and RDEA119 |
| Wang et al. 2018 [56] | FOS, SP1, CDKN1A, CALCR and JUNB | NA |
| Zhai et al. 2020 [57] | NUF2 and FAM83D | NA |
| Shi et al. 2020 [58] | AURKA, BUB1B,CCNB2, CDK1, CDT1, HJURP, KIF20A, KIF2C, KIF4A, MELK, TPX2, and UBE2C | NA |
| Li et al. 2021 [59] | CD3D, CD3E, CD3G, FYN, GRAP2 and ITK. | NA |
| Liu et al. 2020 [60] | CDK1, CDC20, CCNA2, CCNB1, CCNB2, BUB1, BUB1B, CDCA8, KIF11, and TOP2A. | NA |
| Wu et al. 2020 [61] | ESR1, FOXA1, GATA3, PGR, CCND1, GREB1, AR, TFF1, NRIP1 and KRT18, | NA |
| Lou et al. 2018 [62] | MAPK1 and PRDM10 | NA |
| Peng et al. 2020 [63] | SMURF1, BTRC, and TP53 | Yes, Index2 |
| Wei et al. 2021 [64] | CCNB1, CDK1, TOP2A, MKI67, TTK, CCNA2, BUB1, and PLK1 | NA |
| Wang et al. 2020 [65] | CCNE1, CENPN, CHEK1, PLK1, DSCC1, FAM64A, UBE2C, and UBE2T | NA |
| Takashi et al. 2020 [66] | TP53, EGFR, CTNNB1, ERBB2, and HSPB1 | NA |
| He et al. 2015 [67] | DUSP1, MYEOV2, UQCRQ | NA |
| Cao et al. 2020 [68] | HSPB1, TPX2, and IFI16 | NA |
| Hong et al. 2021 [69] | CCNB2, CCNB1, CDC20, PTTG1, BUB1B, TTK and CCNE2 | NA |
| Qin et al. 2016 [70] | DUSP8, FSTL3, TUBA1C, KLF6, EIF3B, UBR2, KIF20A, PTPRK, ZSCAN20, DEPDC1, UNG, and AURKA | NA |
| Tian et al. 2018 [71] | MAGI2-AS3, GGTA1P, NAP1L2, CRABP2, SYNPO2, MKI67, COL4A6 | NA |
| Yuan et al. 2018 [72] | CDC25A, CCNE2, STMN1 and CCNA2. | NA |
| Bai et al. 2020 [73] | STAT1, STAT2, STAT3, STAT6, XBP1, BCL2L1, CYB5D2, ESCO2 | NA |
| Wu et al. 2012 [74] | MCM3, MCM7, BCL2, and TGFB2 | NA |
| Yuan et al. 2021 [75] | CDK1, CCNB1, CCNA2, CDC20, TOP2A, CCNB2, MAD2L1, BUB1, KIF11, RRM2, ESR1 and IGF1 | NA |
| XIAO et al. 2021 [76] | CCNB1, NCAPG, MCM4 and RRM2 | NA |
| Deng et al. 2019 [77] | CDK1, CCNA2, TOP2A, CCNB1, KIF11, and MELK | NA |
| ZHENG et al. 2017 [78] | CAPG, SPI1, LEF1, PBX3, TCF7L2, PLAGL1, EGFR and SYK | NA |
|  |  |  |
| At least 5 articles | KIF11, RRM2, BUB1, CDC20, FOS, FN1, BUB1B, CCNB2, CCNA2, CDK1, TOP2A, CCNB1, EGFR |  |
| At least 7 articles | FN1, BUB1B, CCNB2, CCNA2, CDK1, TOP2A, CCNB1 |  |
| At least 8 articles | CCNA2, CDK1, TOP2A, CCNB1 |  |
| At least 9 articles | CDK1, TOP2A, CCNB1 |  |
| At least 10 articles | CCNB1 |  |

**Index1:** CLADRIBINE, GALLIUM, NITRATE, MOTEXAFIN, GADOLINIUM, HYDROXYUREA, CLOFARABINE, GEMCITABINE, FLUDARABINE, PHOSPHATE, TRIAPINE, FLUDARABINE, DINACICLIB, ALVOCIDIB, Roniciclib, AT-7519, AZD-5438, TG-02, CHEMBL1236539, RG-547, SELICICLIB, SURAMIN, CORDYCEPIN, GENISTEIN

**Index2:** CHEMBL1235116, ASPIRIN, Lesogaberan, A-443654, CHEMBL524266, CHEMBL428963, ALSTERPAULLONE, CHEMBL156987, CHEMBL227381, CHEMBL428462, CHEMBL259850, CHEMBL456218, CHEMBL1082152, STAUROSPORINE, CHEMBL1082552, CHEMBL259833, CHEMBL1230989, ARSENIC TRIOXIDE, TRETINOIN, WORTMANNIN, SF-1126, ISOPRENALINE

**References**

1. Chand Y, Alam MA. Network biology approach for identifying key regulatory genes by expression based study of breast cancer. Bioinformation. 2012; 8(23):1132-8. 10.6026/97320630081132 PMID: 23275709

2. Chen J, Liu C, Cen J, Liang T, Xue J, Zeng H, et al. KEGG-expressed genes and pathways in triple negative breast cancer: Protocol for a systematic review and data mining. Medicine (Baltimore). 2020; 99(18):e19986. 10.1097/MD.0000000000019986 PMID: 32358373

3. Chuan T, Li T, Yi C. Identification of CXCR4 and CXCL10 as Potential Predictive Biomarkers in Triple Negative Breast Cancer (TNBC). Med Sci Monit. 2020; 26:e918281. 10.12659/MSM.918281 PMID: 31924747

4. Dashti S, Taheri M, Ghafouri-Fard S. An in-silico method leads to recognition of hub genes and crucial pathways in survival of patients with breast cancer. Sci Rep. 2020; 10(1):18770. 10.1038/s41598-020-76024-2 PMID: 33128008

5. Dong H, Zhang S, Wei Y, Liu C, Wang N, Zhang P, et al. Bioinformatic analysis of differential expression and core GENEs in breast cancer. Int J Clin Exp Pathol. 2018; 11(3):1146-56 PMID: 31938209

6. Hao M, Liu W, Ding C, Peng X, Zhang Y, Chen H, et al. Identification of hub genes and small molecule therapeutic drugs related to breast cancer with comprehensive bioinformatics analysis. PeerJ. 2020; 8:e9946. 10.7717/peerj.9946 PMID: 33083112

7. Wenners A, Hartmann F, Jochens A, Roemer AM, Alkatout I, Klapper W, et al. Stromal markers AKR1C1 and AKR1C2 are prognostic factors in primary human breast cancer. Int J Clin Oncol. 2016; 21(3):548-56. 10.1007/s10147-015-0924-2 PMID: 26573806

8. Luker KE, Pica CM, Schreiber RD, Piwnica-Worms D. Overexpression of IRF9 confers resistance to antimicrotubule agents in breast cancer cells. Cancer Res. 2001; 61(17):6540-7 PMID: 11522652

9. Marino N, Collins JW, Shen C, Caplen NJ, Merchant AS, Gokmen-Polar Y, et al. Identification and validation of genes with expression patterns inverse to multiple metastasis suppressor genes in breast cancer cell lines. Clin Exp Metastasis. 2014; 31(7):771-86. 10.1007/s10585-014-9667-0 PMID: 25086928

10. Zhang Y, Yu C. Prognostic characterization of OAS1/OAS2/OAS3/OASL in breast cancer. BMC Cancer. 2020; 20(1):575. 10.1186/s12885-020-07034-6 PMID: 32560641

11. Sutherland R, Meeson A, Lowes S. Solute transporters and malignancy: establishing the role of uptake transporters in breast cancer and breast cancer metastasis. Cancer Metastasis Rev. 2020; 39(3):919-32. 10.1007/s10555-020-09879-6 PMID: 32388639

12. Lo Nigro C, Monteverde M, Lee S, Lattanzio L, Vivenza D, Comino A, et al. NT5E CpG island methylation is a favourable breast cancer biomarker. Br J Cancer. 2012; 107(1):75-83. 10.1038/bjc.2012.212 PMID: 22653144

13. Fiorillo M, Sotgia F, Sisci D, Cappello AR, Lisanti MP. Mitochondrial "power" drives tamoxifen resistance: NQO1 and GCLC are new therapeutic targets in breast cancer. Oncotarget. 2017; 8(12):20309-27. 10.18632/oncotarget.15852 PMID: 28411284

14. Kim DW, Cho JY. NQO1 is Required for beta-Lapachone-Mediated Downregulation of Breast-Cancer Stem-Cell Activity. Int J Mol Sci. 2018; 19(12). 10.3390/ijms19123813 PMID: 30513573

15. Makhoul I, Todorova VK, Siegel ER, Erickson SW, Dhakal I, Raj VR, et al. Germline Genetic Variants in TEK, ANGPT1, ANGPT2, MMP9, FGF2 and VEGFA Are Associated with Pathologic Complete Response to Bevacizumab in Breast Cancer Patients. PLoS One. 2017; 12(1):e0168550. 10.1371/journal.pone.0168550 PMID: 28045923

16. Yuan CL, Jiang XM, Yi Y, E JF, Zhang ND, Luo X, et al. Identification of differentially expressed lncRNAs and mRNAs in luminal-B breast cancer by RNA-sequencing. BMC Cancer. 2019; 19(1):1171. 10.1186/s12885-019-6395-5 PMID: 31795964

17. Yan LR, Wang A, Lv Z, Yuan Y, Xu Q. Mitochondria-related core genes and TF-miRNA-hub mrDEGs network in breast cancer. Biosci Rep. 2021; 41(1). 10.1042/BSR20203481 PMID: 33439992

18. Amjad E, Asnaashari S, Sokouti B, Dastmalchi S. Systems biology comprehensive analysis on breast cancer for identification of key gene modules and genes associated with TNM-based clinical stages. Sci Rep. 2020; 10(1):10816. 10.1038/s41598-020-67643-w PMID: 32616754

19. Wang Y, Xu H, Zhu B, Qiu Z, Lin Z. Systematic identification of the key candidate genes in breast cancer stroma. Cell Mol Biol Lett. 2018; 23:44. 10.1186/s11658-018-0110-4 PMID: 30237810

20. Liu X, Ma Y, Yang W, Wu X, Jiang L, Chen X. Identification of therapeutic targets for breast cancer using biological informatics methods. Mol Med Rep. 2015; 12(2):1789-95. 10.3892/mmr.2015.3565 PMID: 25824986

21. Zhang M, Gao CE, Li WH, Yang Y, Chang L, Dong J, et al. Microarray based analysis of gene regulation by mesenchymal stem cells in breast cancer. Oncol Lett. 2017; 13(4):2770-6. 10.3892/ol.2017.5776 PMID: 28454465

22. Yang X, Hu Q, Hu LX, Lin XR, Liu JQ, Lin X, et al. miR-200b regulates epithelial-mesenchymal transition of chemo-resistant breast cancer cells by targeting FN1. Discov Med. 2017; 24(131):75-85 PMID: 28972876

23. Dierssen-Sotos T, Palazuelos-Calderon C, Jimenez-Moleon JJ, Aragones N, Altzibar JM, Castano-Vinyals G, et al. Reproductive risk factors in breast cancer and genetic hormonal pathways: a gene-environment interaction in the MCC-Spain project. BMC Cancer. 2018; 18(1):280. 10.1186/s12885-018-4182-3 PMID: 29530003

24. He N, Zheng H, Li P, Zhao Y, Zhang W, Song F, et al. miR-485-5p binding site SNP rs8752 in HPGD gene is associated with breast cancer risk. PLoS One. 2014; 9(7):e102093. 10.1371/journal.pone.0102093 PMID: 25003827

25. Bhar A, Haubrock M, Mukhopadhyay A, Maulik U, Bandyopadhyay S, Wingender E. Coexpression and coregulation analysis of time-series gene expression data in estrogen-induced breast cancer cell. Algorithms Mol Biol. 2013; 8(1):9. 10.1186/1748-7188-8-9 PMID: 23521829

26. Zhu L, Pan R, Zhou D, Ye G, Tan W. BCL11A enhances stemness and promotes progression by activating Wnt/beta-catenin signaling in breast cancer. Cancer Manag Res. 2019; 11:2997-3007. 10.2147/CMAR.S199368 PMID: 31114347

27. Bao C, Lu Y, Chen J, Chen D, Lou W, Ding B, et al. Exploring specific prognostic biomarkers in triple-negative breast cancer. Cell Death Dis. 2019; 10(11):807. 10.1038/s41419-019-2043-x PMID: 31649243

28. Nishimoto M, Nishikawa S, Kondo N, Wanifuchi-Endo Y, Hato Y, Hisada T, et al. Prognostic impact of TP53INP1 gene expression in estrogen receptor alpha-positive breast cancer patients. Jpn J Clin Oncol. 2019; 49(6):567-75. 10.1093/jjco/hyz029 PMID: 30855679

29. Li MX, Jin LT, Wang TJ, Feng YJ, Pan CP, Zhao DM, et al. Identification of potential core genes in triple negative breast cancer using bioinformatics analysis. Onco Targets Ther. 2018; 11:4105-12. 10.2147/OTT.S166567 PMID: 30140156

30. Lv X, He M, Zhao Y, Zhang L, Zhu W, Jiang L, et al. Identification of potential key genes and pathways predicting pathogenesis and prognosis for triple-negative breast cancer. Cancer Cell Int. 2019; 19:172. 10.1186/s12935-019-0884-0 PMID: 31297036

31. Lu X, Gao C, Liu C, Zhuang J, Su P, Li H, et al. Identification of the key pathways and genes involved in HER2-positive breast cancer with brain metastasis. Pathol Res Pract. 2019; 215(8):152475. 10.1016/j.prp.2019.152475 PMID: 31178227

32. Li Y, Zhou X, Liu J, Yin Y, Yuan X, Yang R, et al. Differentially expressed genes and key molecules of BRCA1/2-mutant breast cancer: evidence from bioinformatics analyses. PeerJ. 2020; 8:e8403. 10.7717/peerj.8403 PMID: 31998560

33. Yang K, Gao J, Luo M. Identification of key pathways and hub genes in basal-like breast cancer using bioinformatics analysis. Onco Targets Ther. 2019; 12:1319-31. 10.2147/OTT.S158619 PMID: 30863098

34. Peng C, Ma W, Xia W, Zheng W. Integrated analysis of differentially expressed genes and pathways in triplenegative breast cancer. Mol Med Rep. 2017; 15(3):1087-94. 10.3892/mmr.2017.6101 PMID: 28075450

35. Wang YW, Zhang W, Ma R. Bioinformatic identification of chemoresistance-associated microRNAs in breast cancer based on microarray data. Oncol Rep. 2018; 39(3):1003-10. 10.3892/or.2018.6205 PMID: 29328395

36. Jin H, Huang X, Shao K, Li G, Wang J, Yang H, et al. Integrated bioinformatics analysis to identify 15 hub genes in breast cancer. Oncol Lett. 2019; 18(2):1023-34. 10.3892/ol.2019.10411 PMID: 31423162

37. Lin Y, Fu F, Lv J, Wang M, Li Y, Zhang J, et al. Identification of potential key genes for HER-2 positive breast cancer based on bioinformatics analysis. Medicine (Baltimore). 2020; 99(1):e18445. 10.1097/MD.0000000000018445 PMID: 31895772

38. Qi L, Zhou B, Chen J, Hu W, Bai R, Ye C, et al. Significant prognostic values of differentially expressed-aberrantly methylated hub genes in breast cancer. J Cancer. 2019; 10(26):6618-34. 10.7150/jca.33433 PMID: 31777591

39. Wang J, Yu H, Yili A, Gao Y, Hao L, Aisa HA, et al. Identification of hub genes and potential molecular mechanisms of chickpea isoflavones on MCF-7 breast cancer cells by integrated bioinformatics analysis. Ann Transl Med. 2020; 8(4):86. 10.21037/atm.2019.12.141 PMID: 32175379

40. Yin X, Wang P, Yang T, Li G, Teng X, Huang W, et al. Identification of key modules and genes associated with breast cancer prognosis using WGCNA and ceRNA network analysis. Aging (Albany NY). 2020; 13(2):2519-38. 10.18632/aging.202285 PMID: 33318294

41. Zhang K, Jiang K, Hong R, Xu F, Xia W, Qin G, et al. Identification and characterization of critical genes associated with tamoxifen resistance in breast cancer. PeerJ. 2020; 8:e10468. 10.7717/peerj.10468 PMID: 33335811

42. Zhu C, Ge C, He J, Zhang X, Feng G, Fan S. Identification of Key Genes and Pathways Associated With Irradiation in Breast Cancer Tissue and Breast Cancer Cell Lines. Dose Response. 2020; 18(2):1559325820931252. 10.1177/1559325820931252 PMID: 32684870

43. Zhang J, Zhou YJ, Yu ZH, Chen AX, Yu Y, Wang X, et al. Identification of core genes and clinical roles in pregnancy-associated breast cancer based on integrated analysis of different microarray profile datasets. Biosci Rep. 2019; 39(6). 10.1042/BSR20190019 PMID: 31171715

44. Lv Q, Liu Y, Huang H, Zhu M, Wu J, Meng D. Identification of Potential Key Genes and Pathways for Inflammatory Breast Cancer Based on GEO and TCGA Databases. Onco Targets Ther. 2020; 13:5541-50. 10.2147/OTT.S255300 PMID: 32606769

45. Zhou J, Yang R. Identification of key pathways and genes shared between Crohn's disease and breast cancer using bioinformatics analysis. Oncol Lett. 2020; 20(4):119. 10.3892/ol.2020.11981 PMID: 32863932

46. Yang M, Li H, Li Y, Ruan Y, Quan C. Identification of genes and pathways associated with MDR in MCF-7/MDR breast cancer cells by RNA-seq analysis. Mol Med Rep. 2018; 17(5):6211-26. 10.3892/mmr.2018.8704 PMID: 29512753

47. Wang Y, Zhang Y, Huang Q, Li C. Integrated bioinformatics analysis reveals key candidate genes and pathways in breast cancer. Mol Med Rep. 2018; 17(6):8091-100. 10.3892/mmr.2018.8895 PMID: 29693125

48. Zhou Z, Wu B, Tang X, Ke R, Zou Q. Comprehensive Analysis of Fibroblast Growth Factor Receptor (FGFR) Family Genes in Breast Cancer by Integrating Online Databases and Bioinformatics. Med Sci Monit. 2020; 26:e923517. 10.12659/MSM.923517 PMID: 32381997

49. Wang Y, Li H, Ma J, Fang T, Li X, Liu J, et al. Integrated Bioinformatics Data Analysis Reveals Prognostic Significance Of SIDT1 In Triple-Negative Breast Cancer. Onco Targets Ther. 2019; 12:8401-10. 10.2147/OTT.S215898 PMID: 31632087

50. Bao S, Chen Y, Yang F, Sun C, Yang M, Li W, et al. Screening and Identification of Key Biomarkers in Acquired Lapatinib-Resistant Breast Cancer. Front Pharmacol. 2020; 11:577150. 10.3389/fphar.2020.577150 PMID: 33013420

51. Zeng F, Fu J, Hu F, Tang Y, Fang X, Zeng F, et al. Identification of key pathways and genes in response to trastuzumab treatment in breast cancer using bioinformatics analysis. Oncotarget. 2018; 9(63):32149-60. 10.18632/oncotarget.24605 PMID: 30181805

52. Liu S, Song A, Wu Y, Yao S, Wang M, Niu T, et al. Analysis of genomics and immune infiltration patterns of epithelial-mesenchymal transition related to metastatic breast cancer to bone. Transl Oncol. 2021; 14(2):100993. 10.1016/j.tranon.2020.100993 PMID: 33333372

53. Zhao CH, Qu L, Zhang H, Qu R. Identification of breast cancer-related circRNAs by analysis of microarray and RNA-sequencing data: An observational study. Medicine (Baltimore). 2019; 98(46):e18042. 10.1097/MD.0000000000018042 PMID: 31725681

54. Fei H, Chen S, Xu C. RNA-sequencing and microarray data mining revealing: the aberrantly expressed mRNAs were related with a poor outcome in the triple negative breast cancer patients. Ann Transl Med. 2020; 8(6):363. 10.21037/atm.2020.02.51 PMID: 32355807

55. Alam MS, Rahaman MM, Sultana A, Wang G, Mollah MNH. Statistics and network-based approaches to identify molecular mechanisms that drive the progression of breast cancer. Comput Biol Med. 2022; 145:105508. 10.1016/j.compbiomed.2022.105508 PMID: 35447458

56. Wang S, Li X, Zhang W, Gao Y, Zhang K, Hao Q, et al. Genome-Wide Investigation of Genes Regulated by ERalpha in Breast Cancer Cells. Molecules. 2018; 23(10). 10.3390/molecules23102543 PMID: 30301189

57. Zhai X, Yang Z, Liu X, Dong Z, Zhou D. Identification of NUF2 and FAM83D as potential biomarkers in triple-negative breast cancer. PeerJ. 2020; 8:e9975. 10.7717/peerj.9975 PMID: 33005492

58. Shi G, Shen Z, Liu Y, Yin W. Identifying Biomarkers to Predict the Progression and Prognosis of Breast Cancer by Weighted Gene Co-expression Network Analysis. Front Genet. 2020; 11:597888. 10.3389/fgene.2020.597888 PMID: 33391348

59. Li L, Huang H, Zhu M, Wu J. Identification of Hub Genes and Pathways of Triple Negative Breast Cancer by Expression Profiles Analysis. Cancer Manag Res. 2021; 13:2095-104. 10.2147/CMAR.S295951 PMID: 33688252

60. Liu S, Liu X, Wu J, Zhou W, Ni M, Meng Z, et al. Identification of candidate biomarkers correlated with the pathogenesis and prognosis of breast cancer via integrated bioinformatics analysis. Medicine (Baltimore). 2020; 99(49):e23153. 10.1097/MD.0000000000023153 PMID: 33285689

61. Wu JR, Zhao Y, Zhou XP, Qin X. Estrogen receptor 1 and progesterone receptor are distinct biomarkers and prognostic factors in estrogen receptor-positive breast cancer: Evidence from a bioinformatic analysis. Biomed Pharmacother. 2020; 121:109647. 10.1016/j.biopha.2019.109647 PMID: 31733575

62. Lou W, Liu J, Ding B, Xu L, Fan W. Identification of chemoresistance-associated miRNAs in breast cancer. Cancer Manag Res. 2018; 10:4747-57. 10.2147/CMAR.S172722 PMID: 30425571

63. Peng Z, Xu B, Jin F. Circular RNA hsa_circ_0000376 Participates in Tumorigenesis of Breast Cancer by Targeting miR-1285-3p. Technol Cancer Res Treat. 2020; 19:1533033820928471. 10.1177/1533033820928471 PMID: 32462972

64. Wei LM, Li XY, Wang ZM, Wang YK, Yao G, Fan JH, et al. Identification of hub genes in triple-negative breast cancer by integrated bioinformatics analysis. Gland Surg. 2021; 10(2):799-806. 10.21037/gs-21-17 PMID: 33708561

65. Wang Y, Li Y, Liu B, Song A. Identifying breast cancer subtypes associated modules and biomarkers by integrated bioinformatics analysis. Biosci Rep. 2021; 41(1). 10.1042/BSR20203200 PMID: 33313822

66. Takeshita T, Yan L, Peng X, Kimbung S, Hatschek T, Hedenfalk IA, et al. Transcriptomic and functional pathway features were associated with survival after pathological complete response to neoadjuvant chemotherapy in breast cancer. Am J Cancer Res. 2020; 10(8):2555-69 PMID: 32905537

67. He J, Yang J, Chen W, Wu H, Yuan Z, Wang K, et al. Molecular Features of Triple Negative Breast Cancer: Microarray Evidence and Further Integrated Analysis. PLoS One. 2015; 10(6):e0129842. 10.1371/journal.pone.0129842 PMID: 26103053

68. Cao W, Jiang Y, Ji X, Guan X, Lin Q, Ma L. Identification of novel prognostic genes of triple-negative breast cancer using meta-analysis and weighted gene co-expressed network analysis. Ann Transl Med. 2021; 9(3):205. 10.21037/atm-20-5989 PMID: 33708832

69. Hong Z, Wang Q, Hong C, Liu M, Qiu P, Lin R, et al. Identification of Seven Cell Cycle-Related Genes with Unfavorable Prognosis and Construction of their TF-miRNA-mRNA regulatory network in Breast Cancer. J Cancer. 2021; 12(3):740-53. 10.7150/jca.48245 PMID: 33403032

70. Qin J, Chen YH. Molecular-level effects of eribulin and paclitaxel on breast cancer based on differential co-expression network analysis. Genet Mol Res. 2016; 15(2). 10.4238/gmr.15028192 PMID: 27420999

71. Tian T, Gong Z, Wang M, Hao R, Lin S, Liu K, et al. Identification of long non-coding RNA signatures in triple-negative breast cancer. Cancer Cell Int. 2018; 18:103. 10.1186/s12935-018-0598-8 PMID: 30026672

72. Yuan N, Zhang G, Bie F, Ma M, Ma Y, Jiang X, et al. Integrative analysis of lncRNAs and miRNAs with coding RNAs associated with ceRNA crosstalk network in triple negative breast cancer. Onco Targets Ther. 2017; 10:5883-97. 10.2147/OTT.S149308 PMID: 29276392

73. Bai J, Luo Y, Zhang S. Microarray data analysis reveals gene expression changes in response to ionizing radiation in MCF7 human breast cancer cells. Hereditas. 2020; 157(1):37. 10.1186/s41065-020-00151-z PMID: 32883354

74. Wu JZ, Lu P, Liu R, Yang TJ. Transcription regulation network analysis of MCF7 breast cancer cells exposed to estradiol. Asian Pac J Cancer Prev. 2012; 13(8):3681-5. 10.7314/apjcp.2012.13.8.3681 PMID: 23098454

75. Yuan Q, Zheng L, Liao Y, Wu G. Overexpression of CCNE1 confers a poorer prognosis in triple-negative breast cancer identified by bioinformatic analysis. World J Surg Oncol. 2021; 19(1):86. 10.1186/s12957-021-02200-x PMID: 33757543

76. Xiao X, Zhang Z, Luo R, Peng R, Sun Y, Wang J, et al. Identification of potential oncogenes in triple-negative breast cancer based on bioinformatics analyses. Oncol Lett. 2021; 21(5):363. 10.3892/ol.2021.12624 PMID: 33747220

77. Deng JL, Xu YH, Wang G. Identification of Potential Crucial Genes and Key Pathways in Breast Cancer Using Bioinformatic Analysis. Front Genet. 2019; 10:695. 10.3389/fgene.2019.00695 PMID: 31428132

78. Zheng T, Wang A, Hu D, Wang Y. Molecular mechanisms of breast cancer metastasis by gene expression profile analysis. Mol Med Rep. 2017; 16(4):4671-7. 10.3892/mmr.2017.7157 PMID: 28791367
