# Supplementary material for: Integrated bioinformatics and statistical approaches to explore molecular biomarkers for breast cancer diagnosis, prognosis and therapies
Source: PLoS One. 2022 May 26;17(5):e0268967. doi: 10.1371/journal.pone.0268967 (PMC9135200; doi:10.1371/journal.pone.0268967)
Supplement: S2 File — (PDF) [file pone.0268967.s002.pdf]

File S2: Supplementary results

Table S1: List of up and down regulated DEGs and key genes (KGs) marked by bold. adj.P.Value < 0.01 and |logFC| > 1.0 threshold have been considered to extracted the differential expressed genes.

Up regulated DEGs	Down regulated DEGs
MATN3, PIGZ, PDE4DIP, ***SLCO2A1****, SGK1, CLN8, ABLIM2, SEMA3C, YPEL4, C8orf58, SEMA3A, FBXO15, NR2F1, CASP4, KDM7A, HIC2, POLR3E, CCDC74B, C12orf76, CFAP206, CASP5, CDADC1, RELB, C5orf45, OSR2, FBXO16, MESP1, TMEM222, CLDN1, CDK6, TBX6, PCDH9, MYO15B, TCTN1, ZC3H10, GLIPR1L2,* ***BCL11A****, CREB5, NOC2L, RGPD1, CYB5R1, CECR5-AS1,* ***NT5E****, CITED4, C14orf37, EVL, LYRM9, LINC01016, JHDM1D-AS1,* ***ANGPT1****, LOC101929147.*	*MARCO, CDCA5, HRASLS2, CTSO, AQP3, FMOD, SORCS2, TRAPPC13, FAM105A, NEURL1, AKR1C3, RFC3, SLIT2, GSE1, HSH2D, TLE2, ANKRD1, CDCA3, CAPG, RASSF2,* ***TP53INP1****, CSTA, MPP1, RARRES3, AQP1, TSPAN2, IFI6, CALHM2, LPAR5, GJB2,* ***NQO1****, EPB41L5, GINS1, EGR1, KIAA0408, PLS3, HOPX, APOL3, PLXNA2, PCDH7, C4BPB, ST6GALNAC5, ZNF385B, SPEG, NUP62CL, PLCH1, DTX3L, IFIT3, AHRR, SESN3, GIMAP2, MELTF, KDELC2, BST2, TIMP3, CDH24, PRUNE2, HPN, METTL7A, JADE2, CARD6, EP400, TDRD10, PODNL1, IFI44L, CCNA1, DHRS2, FAXDC2, DGKH, FOLR1, NEMP1, SPRED1, LDHD, STAT1,* ***OAS1****, RACGAP1P,* ***IRF9****, HES1, PARP9, IFITM1, CITED1, SLC6A12, B3GNT3, NOSTRIN, TDO2, PLAT, TAGLN, FOXN3, CA2, PSMD9,* ***FN1****, SAMD9, COCH, EPB41L1, TRIM14, PML,* ***AKR1C1****, BHLHE40, IFI27, TRIP13, PLEKHS1, CENPJ, RNF39, RSAD2, PDE4A, GLS2, DDX60,* ***OAS3****, OAS2, REC8, TPP1, PMEPA1, ADCY7, CEP128, UNC13A, SECTM1, GLDN, LINC00933, SLC20A2, TRANK1,* ***HPGD****, ACTR3C,* ***ATF6B****, CENPN, COQ2, RAB3B, LINC01224, KNDC1, LNX2, PABPC4L, SLC16A7, C2, FAM101A, DNAH2, MBD3, SHISA2, TDRD12, ZMAT1.*

Bold indicates key genes (KGs).

Table S2: list of 13 identified key genes (kg) with gene name, logFC, p-value, and adjusted p-value.

Gene.symbol	Gene name	logFC	P.Value	adj.P.Val
AKR1C1	aldo-keto reductase family 1 member C1	-2.908633	4.16E-10	4.28E-06
FN1	fibronectin 1	-2.480665	1.71E-06	0.001001
OAS3	2'-5'-oligoadenylate synthetase 3	-2.355372	1.62E-08	4.75E-05
IRF9	interferon regulatory factor 9	-2.331889	8.43E-09	3.15E-05
HPGD	hydroxyprostaglandin dehydrogenase 15-(NAD)	-1.956437	2.15E-09	1.36E-05
OAS1	2'-5'-oligoadenylate synthetase 1	-1.76677	7.49E-05	0.007712
SLCO2A1	solute carrier organic anion transporter family member 2A1	1.72759	5.94E-05	0.006722
ATF6B	activating transcription factor 6 beta	-1.68305	0.000115	0.009925
NT5E	5'-nucleotidase ecto	1.670646	8.56E-06	0.002494
BCL11A	B-cell CLL/lymphoma 11A	1.636371	1.30E-05	0.003059
NQO1	NAD(P)H quinone dehydrogenase 1	-1.577233	5.89E-05	0.006702
ANGPT1	angiopoietin 1	1.55281	2.12E-07	0.000277
TP53INP1	tumor protein p53 inducible nuclear protein 1	-1.517725	6.24E-07	0.000562

S3 Table Gene Ontology (GO) and Kyoto Encyclopedia of Genes and Genomes (KEGG) analysis of the differentially expressed genes (DEGs).

Terms/Functions	Count	PValue	Genes
**GO functional analysis**			
**Down regulated DEGs**			
**GO Terms of Biological Process (BP)**			
GO:0060337~type I interferon signaling pathway	12	8.41E-13	*BST2*, EGR1, *IFITM1*, *IFI27*, *RSAD2*, ***OAS1***, STAT1, OAS2, ***OAS3***, *IFI6*, ***IRF9***, *IFIT3*
GO:0051607~defense response to virus	12	2.72E-08	*BST2*, *IFITM1*, *RSAD2*, ***OAS1***, STAT1, OAS2, ***OAS3***, *DDX60*, *IFI44L*, ***IRF9***, *PML*, *IFIT3*
GO:0009615~response to virus	8	1.38E-05	*BST2*, *IFITM1*, *RSAD2*, ***OAS1***, OAS2, ***OAS3***, *DDX60*, *IFIT3*
GO:0060333~interferon-gamma-mediated signaling pathway	6	1.53E-04	***OAS1***, STAT1, OAS2, ***OAS3***, ***IRF9***, *PML*, ***ATF6B***
GO:0045071~negative regulation of viral genome replication	5	1.86E-04	*BST2*, *IFITM1*, *RSAD2*, ***OAS1***, ***OAS3***
GO:0006302~double-strand break repair	5	0.00127	*DTX3L*, *CDCA5*, *TRIP13*, *PARP9*, *REC8*
GO:0035456~response to interferon-beta	3	0.00176	*BST2*, *IFITM1*, STAT1
GO:0034097~response to cytokine	4	0.0061	*CITED1*, *IFI27*, STAT1, *PML*
GO:0007141~male meiosis I	3	0.00719	*CCNA1*, *TRIP13*, *REC8*
GO:0034341~response to interferon-gamma	3	0.01261	*BST2*, *IFITM1*, *CITED1*
GO:0060700~regulation of ribonuclease activity	2	0.01424	***OAS1***, ***OAS3***
GO:0007584~response to nutrient	4	0.01597	***NQO1***, STAT1, *AKR1C3*, *C2*
GO:0003340~negative regulation of mesenchymal to epithelial transition involved in metanephros morphogenesis	2	0.02129	*CITED1*, STAT1
GO:0003091~renal water homeostasis	3	0.02184	*ADCY7*, *AQP3*, *AQP1*
GO:0008285~negative regulation of cell proliferation	8	0.02361	*IFITM1*, *RARRES3*, ***TP53INP1***, *NEURL1*, *SPEG*, *DHRS2*, *PML*, *IFIT3*
GO:0032355~response to estradiol	4	0.02744	*MBD3*, ***NQO1***, *GJB2*, *IFI27*
GO:0015670~carbon dioxide transport	2	0.02828	*CA2*, *AQP1*
GO:0071395~cellular response to jasmonic acid stimulus	2	0.02828	***AKR1C1***, *AKR1C3*
GO:0046683~response to organophosphorus	2	0.02828	*RFC3*, ***AKR1C1***
GO:0030336~negative regulation of cell migration	4	0.03063	*BST2*, *IFITM1*, ***TP53INP1***, *SLIT2*
GO:0051902~negative regulation of mitochondrial depolarization	2	0.03523	*HSH2D*, *IFI6*
GO:0071504~cellular response to heparin	2	0.03523	EGR1, *SLIT2*
GO:0010756~positive regulation of plasminogen activation	2	0.03523	*HPN*, *MELTF*
GO:0061626~pharyngeal arch artery morphogenesis	2	0.04897	*HES1*, *FOLR1*
GO:0044598~doxorubicin metabolic process	2	0.05577	***AKR1C1***, *AKR1C3*

GO:0044597~daunorubicin metabolic process	2	0.05577	***AKR1C1***, *AKR1C3*
GO:0048146~positive regulation of fibroblast proliferation	3	0.05697	***FN1***, *PML*, *AQP1*
GO:1901796~regulation of signal transduction by p53 class mediator	4	0.05928	*MBD3*, *RFC3*, ***TP53INP1***, *PML*
GO:0055114~oxidation-reduction process	9	0.06191	***NQO1***, ***HPGD***, *SESN3*, *TDO2*, *FAXDC2*, ***AKR1C1***, *LDHD*, *AKR1C3*, *DHRS2*
GO:0042448~progesterone metabolic process	2	0.06252	***AKR1C1***, *AKR1C3*
GO:0035457~cellular response to interferon-alpha	2	0.06252	***OAS1***, *IFIT3*
GO:0042981~regulation of apoptotic process	5	0.06656	EGR1, STAT1, *GLS2*, ***TP53INP1***, *CARD6*
GO:0035455~response to interferon-alpha	2	0.06923	*BST2*, *IFITM1*
GO:0035385~Roundabout signaling pathway	2	0.08249	*SLIT2*, *FMOD*
GO:0042574~retinal metabolic process	2	0.08249	***AKR1C1***, *AKR1C3*
GO:0048846~axon extension involved in axon guidance	2	0.08249	*SLIT2*, *FMOD*
GO:0043517~positive regulation of DNA damage response, signal transduction by p53 class mediator	2	0.08249	*SPRED1*, *ANKRD1*
GO:0006164~purine nucleotide biosynthetic process	2	0.08249	***OAS1***, OAS2
GO:0032868~response to insulin	3	0.08297	EGR1, *CITED1*, *SESN3*
GO:0045087~innate immune response	7	0.08806	*BST2*, *MARCO*, *TRIM14*, *C4BPB*, *DDX60*, *PML*, *C2*
GO:0006693~prostaglandin metabolic process	2	0.08905	***HPGD***, *AKR1C3*
GO:0009992~cellular water homeostasis	2	0.08905	*AQP3*, *AQP1*
GO:0015793~glycerol transport	2	0.08905	*AQP3*, *AQP1*
GO:0030855~epithelial cell differentiation	3	0.08941	*TAGLN*, ***AKR1C1***, *TPP1*
GO:0071361~cellular response to ethanol	2	0.09557	***TP53INP1***, *ADCY7*
GO:0007064~mitotic sister chromatid cohesion	2	0.09557	*CDCA5*, *REC8*
**GO Terms of Cellular Component (CC)**			
GO:0005737~cytoplasm	57	4.91E-05	*RAB3B*, *CITED1*, *GIMAP2*, *PLAT*, *AQP3*, *IFI44L*, *IFIT3*, *AQP1*, *CEP128*, *PSMD9*, *SPRED1*, *PODNL1*, *RASSF2*, *SESN3*, *CA2*, *ANKRD1*, *PLS3*, *UNC13A*, *DTX3L*, *HRASLS2*, *PARP9*, ***OAS1***, OAS2, ***OAS3***, *AHRR*, *TRIM14*, ***IRF9***, *DGKH*, *CSTA*, *TAGLN*, *DNAH2*, *SAMD9*, ***HPGD***, *CDCA5*, *PRUNE2*, *CAPG*, *DDX60*, *EPB41L5*, *EPB41L1*, ***TP53INP1***, *RNF39*, *HES1*, *SLIT2*, *METTL7A*, *APOL3*, *GINS1*, *MBD3*, EGR1, ***NQO1***, STAT1, *AKR1C3*, *DHRS2*, *HOPX*, *PML*, *BST2*, *GJB2*, *PLCH1*
GO:0005578~proteinaceous extracellular matrix	7	0.009721	*PODNL1*, ***FN1***, TIMP3, *SLIT2*, *FMOD*, *GLDN*, *COCH*
GO:0005911~cell-cell junction	5	0.029262	*CDCA3*, *EPB41L1*, *HPN*, *CDH24*,

			AQP3
GO:0016323~basolateral plasma membrane	5	0.033759	***HPGD***, *CA2*, *AQP3*, *FOLR1*, *AQP1*
GO:0005615~extracellular space	16	0.037725	*CSTA*, *TLE2*, ***FN1***, *SECTM1*, *PLAT*, *C4BPB*, *GLDN*, *C2*, *CTSO*, *CA2*, ***OAS3***, TIMP3, *SLIT2*, *MELTF*, *FMOD*
GO:0031012~extracellular matrix	6	0.050375	*CSTA*, ***FN1***, TIMP3, *PLAT*, *FMOD*, *COCH*
GO:0005887~integral component of plasma membrane	16	0.053921	*MPP1*, *SLC20A2*, *PCDH7*, *HPN*, *SLC6A12*, *AQP3*, *AQP1*, *BST2*, *MARCO*, *CALHM2*, *PLXNA2*, *B3GNT3*, *SLC16A7*, *TSPAN2*, *MELTF*, *FOLR1*
GO:0070062~extracellular exosome	27	0.05531	*RAB3B*, *CSTA*, *SLC20A2*, ***HPGD***, *HPN*, *SECTM1*, *PLAT*, *CAPG*, *JADE2*, *COCH*, *C2*, *AQP1*, *ACTR3C*, *CA2*, TIMP3, *SLIT2*, *METTL7A*, ***NQO1***, ***AKR1C1***, *AKR1C3*, ***FN1***, *DHRS2*, *BST2*, *TPP1*, *MELTF*, *FOLR1*
GO:0005811~lipid particle	3	0.07331	*RSAD2*, *GIMAP2*, *METTL7A*
GO:0005829~cytosol	30	0.07858	*RAB3B*, *CDCA3*, *CITED1*, ***HPGD***, *CDCA5*, *IFIT3*, *PSMD9*, *SPRED1*, *TDO2*, *EPB41L1*, *CA2*, ***TP53INP1***, *ANKRD1*, *PDE4A*, ***NQO1***, *DTX3L*, STAT1, ***AKR1C1***, *AKR1C3*, *PARP9*, *PML*, *CCNA1*, *HSH2D*, ***OAS1***, OAS2, ***OAS3***, *CENPJ*, *CENPN*, *PLCH1*, ***IRF9***
GO:0045177~apical part of cell	3	0.091261	*CA2*, *PLAT*, *AQP1*
**GO Terms of Molecular Function (MF)**			
GO:0001730~2'-5'-oligoadenylate synthetase activity	3	3.28E-04	***OAS1***, OAS2, ***OAS3***
GO:0003725~double-stranded RNA binding	4	0.010666	***OAS1***, OAS2, ***OAS3***, *DDX60*
GO:0016779~nucleotidyltransferase activity	3	0.017174	***OAS1***, OAS2, ***OAS3***
GO:0004871~signal transducer activity	6	0.01849	*BST2*, *UNC13A*, STAT1, *SECTM1*, *PLCH1*, *APOL3*
GO:0047086~ketosteroid monooxygenase activity	2	0.022227	***AKR1C1***, *AKR1C3*
GO:0047718~indanol dehydrogenase activity	2	0.022227	***AKR1C1***, *AKR1C3*
GO:0018636~phenanthrene 9,10-monooxygenase activity	2	0.029526	***AKR1C1***, *AKR1C3*
GO:0047115~trans-1,2-dihydrobenzene-1,2-diol dehydrogenase activity	2	0.029526	***AKR1C1***, *AKR1C3*
GO:0004032~alditol:NADP+ 1-oxidoreductase activity	2	0.051101	***AKR1C1***, *AKR1C3*
GO:0016655~oxidoreductase activity, acting on NAD(P)H, quinone or similar compound as acceptor	2	0.058187	***AKR1C1***, *AKR1C3*
GO:0016491~oxidoreductase activity	5	0.062918	***HPGD***, *SESN3*, ***AKR1C1***, *AKR1C3*, *DHRS2*
GO:0048495~Roundabout binding	2	0.06522	*SLIT2*, *FMOD*
GO:0001191~transcriptional repressor	2	0.086008	*BHLHE40*, *AHRR*

activity, RNA polymerase II transcription factor binding			
GO:0015254~glycerol channel activity	2	0.086008	*AQP3*, *AQP1*
**Up regulated DEGs**			
**GO Terms of BP**			
GO:0010977~negative regulation of neuron projection development	4	1.04E-04	***BCL11A***, *SEMA3A*, *NR2F1*, *TBX6*
GO:0060666~dichotomous subdivision of terminal units involved in salivary gland branching	2	0.01038	*SEMA3C*, *SEMA3A*
GO:0045944~positive regulation of transcription from RNA polymerase II promoter	7	0.014821	*OSR2*, *MESP1*, ***BCL11A***, *CITED4*, *NR2F1*, *TBX6*, *RELB*, ***SLCO2A1***
GO:0000122~negative regulation of transcription from RNA polymerase II promoter	6	0.015973	*OSR2*, ***BCL11A***, *NR2F1*, *TBX6*, *NOC2L*, *RELB*, ***NT5E***
GO:0006351~transcription, DNA-templated	10	0.016336	*HIC2*, *ABLIM2*, ***BCL11A***, *POLR3E*, *CITED4*, *NR2F1*, *TBX6*, *NOC2L*, ***NT5E***, *KDM7A*, *RELB*, ***ANGPT1***
GO:0006915~apoptotic process	5	0.029434	*CASP5*, *SEMA3A*, *CASP4*, *NOC2L*, *SGK1*, ***ANGPT1***,
GO:0010633~negative regulation of epithelial cell migration	2	0.030825	*SEMA3A*, *EVL*
GO:0023019~signal transduction involved in regulation of gene expression	2	0.038888	*MESP1*, *TBX6*
GO:0007411~axon guidance	3	0.043237	*SEMA3C*, *SEMA3A*, *EVL*
GO:0048843~negative regulation of axon extension involved in axon guidance	2	0.052842	*SEMA3C*, *SEMA3A*
GO:0071526~semaphorin-plexin signaling pathway	2	0.066599	*SEMA3C*, *SEMA3A*
GO:0050919~negative chemotaxis	2	0.068548	*SEMA3C*, *SEMA3A*
GO:0042981~regulation of apoptotic process	3	0.072523	*CASP5*, *CASP4*, *SGK1*
GO:0001755~neural crest cell migration	2	0.089732	*SEMA3C*, *SEMA3A*
GO:0045893~positive regulation of transcription, DNA-templated	4	0.091214	*OSR2*, *MESP1*, *KDM7A*, *CREB5*
**GO Terms of CC**			
GO:0072557~IPAF inflammasome complex	2	0.010928	*CASP5*, *CASP4*
GO:0097169~AIM2 inflammasome complex	2	0.013099	*CASP5*, *CASP4*
GO:0072559~NLRP3 inflammasome complex	2	0.017428	*CASP5*, *FN1*
**GO Terms of MF**			
GO:0097153~cysteine-type endopeptidase activity involved in apoptotic process	2	0.02663	*CASP5*, *FN1*
GO:0038191~neuropilin binding	2	0.030665	*SEMA3C*, *SEMA3A*
GO:0030215~semaphorin receptor binding	2	0.046645	*SEMA3C*, *SEMA3A*
GO:0045499~chemorepellent activity	2	0.054538	*SEMA3C*, *SEMA3A*
**KEGG enrichment analysis**			
**Down regulated DEGs**			
hsa05164:Influenza A	7	0.002058	*RSAD2*, ***OAS1***, STAT1, OAS2, ***OAS3***, *PML*, ***IRF9***
hsa05168:Herpes simplex infection	6	0.012895	***OAS1***, STAT1, OAS2, ***OAS3***, *PML*,

			ATF6B, ***IRF9***
hsa04964:Proximal tubule bicarbonate reclamation	3	0.013288	*CA2*, *GLS2*, *AQP1*
hsa05162:Measles	5	0.018835	***OAS1***, STAT1, OAS2, ***OAS3***, ***IRF9***
hsa05160:Hepatitis C	5	0.018835	***OAS1***, STAT1, OAS2, ***OAS3***, ***IRF9***
hsa00130:Ubiquinone and other terpenoid-quinone biosynthesis	2	0.081617	***NQO1***, *COQ2*
hsa04610:Complement and coagulation cascades	3	0.098614	*PLAT*, *C4BPB*, *C2*
hsa04976:Bile secretion	3	0.098614	*CA2*, *ADCY7*, *AQP1*
**Up regulated DEGs**			
hsa04360:Axon guidance	3	0.026619	*ABLIM2*, *SEMA3C*, *SEMA3A*
hsa04151:PI3K-Akt signaling pathway	4	0.030138	*CDK6*, ***ANGPT1***, *SGK1*, ***SLCO2A1***, *CREB5*, ***BCL11A***

***Note:** Bold indicates key genes (KGs)
